# Functional analysis of the methylerythritol phosphate pathway terminal enzymes IspG and IspH from *Zymomonas mobilis*

**DOI:** 10.1128/spectrum.04256-23

**Published:** 2024-05-24

**Authors:** Jyotsna Misra, Erin L. Mettert, Patricia J. Kiley

**Affiliations:** 1 Department of Biomolecular Chemistry, University of Wisconsin-Madison, Madison, Wisconsin, USA; 2 DOE Great Lakes Bioenergy Research Center, University of Wisconsin-Madison, Madison, Wisconsin, USA; University of Hong Kong, Hong Kong, Hong Kong

**Keywords:** IspH, IspG, Fe-S cluster stability, isoprenoid synthesis, MEP pathway optimization, IDP, MEcDP, HMBDP, DMADP, oxygen-sensitive Fe-S clusters, alpha proteobacteria

## Abstract

Isoprenoids are a diverse family of compounds that are synthesized from two isomeric compounds, isopentenyl diphosphate and dimethylallyl diphosphate. In most bacteria, isoprenoids are produced from the essential methylerythritol phosphate (MEP) pathway. The terminal enzymes of the MEP pathway IspG and IspH are [4Fe-4S] cluster proteins, and in *Zymomonas mobilis,* the substrates of IspG and IspH accumulate in cells in response to O_2_, suggesting possible lability of their [4Fe-4S] clusters. Here, we show using complementation assays in *Escherichia coli* that even under anaerobic conditions, *Z. mobilis* IspG and IspH are not as functional as their *E. coli* counterparts, requiring higher levels of expression to rescue viability. A deficit of the sulfur utilization factor (SUF) Fe-S cluster biogenesis pathway did not explain the reduced function of *Z. mobilis* IspG and IspH since no improvement in viability was observed in *E. coli* expressing the *Z. mobilis* SUF pathway or having increased expression of the *E. coli* SUF pathway. Complementation of single and double mutants with various combinations of *Z. mobilis* and *E. coli* IspG and IspH indicated that optimal growth required the pairing of IspG and IspH from the same species. Furthermore, *Z. mobilis* IspH conferred an O_2_-sensitive growth defect to *E. coli* that could be partially rescued by co-expression of *Z. mobilis* IspG. *In vitro* analysis showed O_2_ sensitivity of the [4Fe-4S] cluster of both *Z. mobilis* IspG and IspH. Altogether, our data indicate an important role of the cognate protein IspG in *Z. mobilis* IspH function under both aerobic and anaerobic conditions.

**IMPORTANCE:**

Isoprenoids are one of the largest classes of natural products, exhibiting diversity in structure and function. They also include compounds that are essential for cellular life across the biological world. In bacteria, isoprenoids are derived from two precursors, isopentenyl diphosphate and dimethylallyl diphosphate, synthesized primarily by the methylerythritol phosphate pathway. The aerotolerant *Z. mobilis* has the potential for methylerythritol phosphate pathway engineering by diverting some of the glucose that is typically efficiently converted into ethanol to produce isoprenoid precursors to make bioproducts and biofuels. Our data revealed the surprising finding that *Z. mobilis* IspG and IspH need to be co-optimized to improve flux via the methyl erythritol phosphate pathway in part to evade the oxygen sensitivity of IspH.

## INTRODUCTION

Isoprenoids comprise a chemically diverse family of natural products that have various functions throughout the biological world (1–3). Isoprenoids also have commercial value because of their use as colorants, flavoring agents, vitamins, and fragrances and their potential to be used as alternative fuel sources (4, 5). Isoprenoid precursors, isopentenyl diphosphate (IDP) and dimethylallyl diphosphate (DMADP), are synthesized from one of two conserved pathways. The methylerythritol phosphate (MEP) pathway is found in eubacteria, green algae, and plant plastids (6), whereas the mevalonate (MVA) pathway is found in *Streptomyces,* Archaea, plant cytosol, and other eukaryotes (4). Recent studies have focused on the MEP pathway as a possible platform for the engineering of isoprenoid compounds (7).

Studies of *Zymomonas mobilis,* a Gram-negative*,* alpha-proteobacterium, are providing new insights into the control of flux through the MEP pathway that may prove useful in expanding use of this ethanol-producing organism for the production of isoprenoid-based biofuels (8, 9). *Z. mobilis* naturally makes large amounts of hopanoids, a class of isoprenoids (10), and converts 96% of glucose to ethanol by the Entner-Doudoroff glycolytic pathway under anaerobic conditions (11). Thus, *Z. mobilis* is an excellent microbe for engineering the MEP pathway because of its streamlined catabolism and the potential to redeploy more carbon from glucose to optimize IDP and DMADP production (12).

The last two steps of the MEP pathway require the enzymes IspG and IspH, and these have been identified as key metabolic bottlenecks in *Z. mobilis* overexpression studies (8). Mechanistic studies of these enzymes from various sources indicate that IspG catalyzes the reductive ring opening of 2-C-methyl-D-erythritol 2,4-cyclodiphosphate (MEcDP) to 4-hydroxy-3-methyl-but-2-enyl diphosphate (HMBDP) (13). The structure of IspG from *Thermus thermophilus* and *A. aeolicus* reveals that the protein is a dimer, with each monomer coordinating an [4Fe-4S] cluster via a Glu and three Cys residues (14, 15). In contrast, IspH from *Escherichia coli* is a monomer coordinating the [4Fe-4S] cluster via three Cys residues and the substrate HMBDP (16, 17). The reduced cluster promotes the reductive dehydroxylation of HMBDP, generating the isomers IDP and DMADP. However, promiscuous activities of IspH have also been observed. In its reduced [4Fe-4S]^1+^ state, IspH from *Bacillus* sp. N16-5 also catalyzes the conversion of HMBDP to isoprene and DMADP to isoamylene (18). In its oxidized [4Fe-4S]^2+^ state, *E. coli* IspH has also been shown to catalyze the conversion of acetylenes to ketones and aldehydes via an acetylene hydratase activity (19). These data suggest that the Fe-S cluster redox state is one feature that influences optimal metabolic flux through this pathway.

The Fe-S clusters of these enzymes are generally considered to be O_2_ sensitive since anaerobic conditions are required for isolation of active enzymes (20, 21). Indeed, when *Z. mobilis* was shifted from anaerobic to aerobic growth conditions, the substrates of IspG and IspH, MEcDP, and HMBDP, respectively, accumulated transiently, suggesting that the Fe-S clusters of *Z. mobilis* IspG and IspH are also labile to O_2_. The *suf* operon, encoding the sole Fe-S biogenesis pathway of *Z. mobilis,* was also upregulated in response to O_2_, suggesting an increase in demand for Fe-S cluster biogenesis perhaps to replace or repair O_2_-damaged enzymes (9). In recent work in *E. coli*, the role of heterologous Fe-S biogenesis pathways was investigated and shown to be important in the function of several types of Fe-S cluster enzymes from analogous sources (22). Taken together, these observations suggest that cluster occupancy may also be limiting metabolic flux of the MEP pathway in some circumstances.

Here, we investigate the function of *Z. mobilis* IspG and IspH in *E. coli*, taking advantage of the established genetic tools to study MEP pathway function, which is essential for viability. Since previous studies suggest that the [4Fe-4S] cluster of *Z. mobilis* IspG or IspH could be sensitive to oxygen, and insufficient levels impede metabolic flux (9), we sought to analyze properties of the enzymes that may lead to improved MEP pathway flux in *Z. mobilis*. By comparing the function of *Z. mobilis* IspG and IspH with *E. coli* orthologs under aerobic and anaerobic conditions, we define several features of *Z. mobilis* IspG and IspH that may limit its activity in cells. The stability of the [4Fe-4S] clusters of these enzymes to O_2_ was also examined *in vitro*. We also tested whether elevated expression of either the *E. coli* or *Z. mobilis* SUF Fe-S biogenesis pathway enhanced *Z. mobilis* IspG or IspH activity in *E. coli*. These results indicate that *Z. mobilis* IspG and IspH are not as functional as their *E. coli* counterparts and that *Z. mobilis* IspH imparts an extreme O_2_-sensitive growth phenotype to *E. coli* that cannot be rescued by the SUF Fe-S machinery. These results will guide future engineering strategies of these MEP pathway enzymes in *Z. mobilis*.

## MATERIAL AND METHODS

Bacterial strains and plasmids used in this study are listed in Tables 1 and 2.

**TABLE 1:** Strains

Strain no.	Description	Source
PK13681	BL21 (DE3) Δ*him*::*tet* Δ*iscR*::*kan P_BAD_suf*	Lab collection (23)
PK13654	PK13681 with pPK13650	This study
PK13652	PK13681 with pPK13648	This study
PK13655	PK13681 with pPK13651	This study
BP129	*E. coli putP*::*kan P*_*BAD*_ *yPMD, hPMK, yMVK, ecIDI, lacZYA*	Beatrice Py
PK13556	MG1655 *putP*::*kan P*_*BAD*_ *yPMD, hPMK, yMVK, ecIDI, lacZYA*	This study
PK9012	MG1655 Δ*mutS*::*Tn10* λ*c1857* Δ*cro-bioA*	Lab collection (24)
PK13557	PK9012 *putP*::*kan P*_*BAD*_ *yPMD, hPMK, yMVK, ecIDI, lacZYA*	This study
PK13566	PK13556 Δ*ispH*::*cat*	This study
PK13567	PK13556 ΔispG::*cat*	This study
PK9776	MG1655 Δ*lacY*	Lab collection
PK14463	MG1655 Δ*lacY putP*:: Δ*kan P*_*BAD*_ *yPMD, hPMK, yMVK, ecIDI,* Δ*lacZYA::tet*	This study
PK14464	PK14463 Δ*ispH*::*cat*	This study
PK14478	PK14464 with pPK13573	This study
PK14467	PK14464 with pPK13574	This study
PK14468	PK14464 with pPK13635	This study
PK14465	PK14463 ΔispG::*cat*	This study
PK14473	PK14465 with pPK13575	This study
PK14474	PK14465 with pPK13576	This study
PK14485	PK14465 Δ*ispG*	This study
PK14487	PK14485 ΔispH::*cat*	This study
PK14489	PK14487 with pPK13636	This study
PK14488	PK14487 with pPK13635	This study
PK14490	PK14487 with pPK13985	This study
PK14494	PK14487 with pPK13986	This study
PK14498	PK14464 with pPK13612	This study
PK14481	PK14464 with pPK13616	This study
PK14483	PK14464 with pPK14414	This study
PK14484	PK14465 with pPK13614	This study
PK14486	PK14465 with pPK13618	This study
PK14499	PK14487 with pPK14414	This study
PK16191	MG1655 *kan*-P*_fnr_*-*suf*_*EC*_	This study
PK14842	PK14464 *kan-*P_*fnr*_-*suf*_*EC*_	This study
PK14849	PK14842 with pPK13573	This study
PK14843	PK14842 with pPK13574	This study
PK14844	PK14842 with pPK13635	This study
PK16235	MG1655 *kan*-P*_fnr_*-*suf*_*ZM*_	This study
PK14862	PK14464 *kan*-P*_fnr_*-*suf*_*ZM*_	This study
PK14869	PK14862 with pPK13573	This study
PK14870	PK14862 with pPK13574	This study
PK14871	PK14862 with pPK13635	This study
PK13593	PK13566 with pPK13574	This study
PK14433	PK13566 with pPK14428	This study
PK14440	PK13566 with PPK14408	This study
BTH101		(25)
PK14875	BTH101 with pUT18C-ZIP and pKT25-ZIP	This study
PK14876	BTH101 with pUT18C and pKT25	This study
PK14877	BTH101 with pPK14865 and pPK14868	This study
PK14878	BTH101 with pPK14865 and pPK14867	This study
PK14879	BTH101 with pPK14864 and pPK14866	This study

**TABLE 2:** Plasmids

Plasmid no.	Description	Source
pRL814	Broad host range plasmid	Lab collection (26)
pPK13573	pRL814 with *E. coli ispH*	This study
pPK13574	pRL814 with *Z. mobilis ispH*	This study
pPK13575	pRL814 with *E. coli ispG*	This study
pPK13576	pRL814 with *Z. mobilis ispG*	This study
pPK13635	pRL814 with *Z. mobilis ispH* and *Z. mobilis ispG*	This study
pPK13636	pRL814 with *E. coli ispH* and *E. coli ispG*	This study
pPK13985	pRL814 with *E. coli ispH* and *Z. mobilis ispG*	This study
pPK13986	pRL814 with *Z. mobilis ispH* and *E. coli ispG*	This study
pPK14408	pRL814 with *Z. mobilis ispH*C13A	This study
pPK14428	pRL814 with *Z. mobilis ispH*E127A	This study
pPK13612	pRL814 with *E. coli ispH* Strep-tag II	This study
pPK13614	pRL814 with *E. coli ispG* Strep-tag II	This study
pPK13616	pRL814 with *Z. mobilis ispH* Strep-tag II	This study
pPK13618	pRL814 with *Z. mobilis ispG* Strep-tag II	This study
pPK14414	pRL814 with *Z. mobilis ispH* Strep-tag II and *ispG*	This study
pET11a		Lab collection
pPK13648	pET11a with *E. coli ispH* Strep-tag II	This study
pPK13650	pET11a with *Z. mobilis ispH* Strep-tag II	This study
pPK13651	pET11a with *Z. mobilis ispG* Strep-tag II	This study
pCP20	FLP recombinase	B. L. Wanner (27)
pPKD32	FRT-*cat*-FRT	B. L. Wanner (27)
pKD46	Lambda recombinase	B. L. Wanner (27)
pUT18C		(25)
pKT25		(25)
pUT18-ZIP		(25)
pKT25-ZIP		(25)
pPK14868	pKT25 with *E. coli ispH* fused in frame at the KpnI site	This study
pPK14866	pKT25 with *Z. mobilis ispH* fused in frame at the KpnI site	This study
pPK14865	pUT18C with *E. coli ispG* fused in frame at the KpnI site	This study
pPK14864	pUT18C with *Z. mobilis ispG* fused in frame at the KpnI site	This study
pPK14867	pKT25 with *E. coli ispG* fused in frame at the KpnI site	This study
pPK6980	P_fnr_ plasmid	Lab collection (28)

### Plasmid construction

The coding regions of *ispG* and *ispH* from *E. coli* and *Z. mobilis* were cloned into the broad host range, isopropyl-β-D-thiogalactoside (IPTG)-inducible expression vector, pRL814 (26), for use in complementation assays. First, each coding region was amplified from either *E. coli* MG1655 or *Z. mobilis* ZM4 genomic DNA by PCR and specific primer sets (Table S1). Primer sets 1 (a, b, and c) and 3 (a, b, and c) amplified *E. coli ispH* and *ispG*, respectively. Primer sets 2 (a, b, and c) and 4 (a, b, and c) amplified *Z. mobilis ispH* and *ispG,* respectively. The PCR products were digested with AflII and SalI and ligated into the same sites of pRL814 (26) to yield pPK13575 for *E. coli ispG*, pPK13576 for *Z. mobilis ispG*, pPK13573 for *E. coli ispH*, and pPK13574 for *Z. mobilis ispH* (Table 2). For these expression plasmids, the ribosomal binding sites of *Z. mobilis ispG* and *ispH* were also independently optimized using the Salis algorithm (29), and the analogous optimized sequence was also used for *E. coli ispG* and *ispH*. The ribosomal binding site and adjacent optimized sequence were included in the primers used to amplify *ispH* and *ispG* and are highlighted in bold in the primer sequence (Table S1). To construct plasmids that co-express *ispH* and *ispG, Z. mobilis* and *E. coli ispG* were amplified from pPK13576 and pPK13575, respectively, using primer pair 8a and 8b and primer pair 9a and 9b, respectively. pPK13574 or pPK13573 was amplified with primers 7a and 7b, and the appropriate *ispG* PCR products were inserted 121 nucleotides downstream of *ispH* by Gibson assembly (New England Biolabs [NEB]) to generate plasmids pPK13635 and pPK13636, respectively. For co-expression of *ispH* from *E. coli* and *ispG* from *Z. mobilis, Z. mobilis ispG* from pPK13576 was amplified using primer set 11a and 11b and inserted 121 nucleotides downstream of *E. coli ispH* in pPK13573, amplified using primer set 10a and 10b, to generate pPK13985. In addition, *E. coli ispG* from pPK13575 was amplified using primer set 13a and 13b and inserted 121 nucleotides downstream of *Z. mobilis ispH* in pPK13574, amplified by primer set 12a and 12b, to generate pPK13986. *Z. mobilis* plasmid-encoded IspH variants resulting in a C13A (pPK14408) or E127A (pPK14428) substitution were derived from pPK13574 and were synthesized by GENEWIZ. To generate in-frame C-terminal Strep-tagged variants of IspH and IspG, the DNA sequence encoding Strep-tag II was included in the primer sequence for PCR amplification of *Z. mobilis ispH* (primer set 14a and 14b), *E. coli ispH* (primer set 15a and 15b), *Z. mobilis ispG* (primer set 16a and 16b), *E. coli ispG* (primer set 17a and 17b), and KLD enzyme mix (NEB) was used for assembly to generate plasmids pPK13616, pPK13612, pPK13618, and pPK13614, respectively. Strep-tag II was also added to *Z. mobilis ispH* on plasmid pPK13635 to produce pPK14414. For isolation of protein, *Z. mobilis ispH* with Strep-tag II was constructed from PCR products of *ispH* amplified from pPK13574 using primers 18a and 18b, and pET11a, amplified by primers 19a and 19b, joined by Gibson assembly to generate pPK13650. In a similar way, pET11a expression plasmids bearing either *Z. mobilis ispG* with Strep-tag II (pPK13651) or *E.coli ispH* with Strep-tag II (pPK13648) were constructed using primers 20 and 20b or 21a and 21b, respectively. Plasmid clones were confirmed by DNA sequencing.

### Strain construction

To delete the essential genes *ispH* and *ispG* from *E. coli* MG1655 or its recombineering derivative PK9012 (24), we used an established approach to maintain viability by first P1 transducing into these strains a synthetic operon encoding the eukaryotic mevalonic acid pathway (*yPMD, hPMK, yMVK,* and *ecIDI*), under control of the *E. coli* arabinose-inducible promoter P*_BAD_* and flanked by *kan* and a second copy of *lacZYA* (*kan P_BAD_ yPMD, hPMK, yMVK, ecIDI*, and *lacZYA*) (30) inserted at the *putP* locus from strain BP129 to create PK13556 and PK13557, respectively. Δ*ispH::cat* or Δ*ispG::cat* derivatives of PK13557 were created by electroporation of PCR fragments from amplified FRT-*cat*-FRT from pKD32 (27) with primers containing 5′ tails that are homologous to the sequence flanking the coding region of *ispH* and *ispG* (primer pairs 5a and 5b and 6a and 6b, respectively). Cell viability was maintained by growth on LB media containing 10 mM arabinose to induce MVA^+^ expression and 1 mM mevalonate to bypass the MEP pathway. Mevalonate was made from 0.9 M mevalonolactone (EMD Millipore) by alkaline hydrolysis with 1 M potassium hydroxide. The Δ*ispH::cat* or Δ*ispG::cat* alleles were then P1 transduced to PK13556, resulting in PK13566 (MG1655Δ*ispH::cat putP::kan P_BAD_ yPMD, hPMK, yMVK, ecIDI*, *lacZYA*) and PK13567 (MG1655Δ*ispG::cat putP::kan P_BAD_ yPMD, hPMK, yMVK, ecIDI*, *lacZYA*), respectively.

The *kan* gene from the MVA cassette was then replaced with FRT-*cat*-FRT from pKD32 (27) by PCR amplification and electroporation into PK13556 containing pKD46, and the *cat* gene was subsequently removed with pCP20 to facilitate additional strain modifications (27). To obtain a homogeneous response to IPTG in our expression experiments (31), the *lacZYA* genes were deleted from the MVA cassette by replacing with *tet*. PCR-amplified *tet* from pBR322 using primers containing 5′ tails that are homologous to the sequence flanking *lacZYA* was electroporated into PK14282 containing pKD46. The modified MVA^+^ cassette (*putP*::Δ*kan* P*_BAD_ yPMD, hPMK, yMVK, ecIDI*, Δ*lacZYA::tet*) was then P1 transduced to PK9776 (MG1655 Δ*lacY*) to yield PK14463. The Δ*ispH::cat* and Δ*ispG::cat* alleles from PK13566 and PK13567 were then P1 transduced to PK14463, resulting in PK14464 (MG1655Δ*ispH::cat*Δ*lacY putP*::Δ*kan* P*_BAD_ yPMD, hPMK, yMVK, ecIDI*, Δ*lacZYA::tet*) and PK14465 (MG1655Δ*ispG::cat* Δ*lacY putP::*Δ*kan* P*_BAD_ yPMD, hPMK, yMVK, ecIDI*, Δ*lacZYA::tet*), respectively. Plasmids carrying the *E. coli* or *Z. mobilis ispH* or *Z. mobilis ispHispG* were transformed into PK14464. Plasmids carrying *E. coli* and *Z. mobilis ispG* genes were transformed into PK14465. The vector pRL814 was also transformed into PK14464 and PK14465.

To create the double Δ*ispH*Δ*ispG* mutant, the *cat* gene from Δ*ispG::cat* of PK14465 was removed using pCP20 to generate PK14485 and then subsequently transduced with the Δ*ispH::cat* allele from PK13566 to yield PK14487 (MG1655 Δ*ispG*Δ*ispH::cat* Δ*lacY putP::*Δ*kan* P*_BAD_ yPMD, hPMK, yMVK, ecIDI*, Δ*lacZYA::tet*). Plasmids containing *ispH* and *ispG* genes from *E. coli* and *Z. mobilis* were transformed in PK14487.

Strains overexpressing the SUF pathway from either *E. coli* or *Z. mobilis* were constructed as follows. A previously characterized derivative of the *fnr* promoter, *kan-P_fnr_* from pPK6980 (28), was used to drive constitutive, moderate expression of *suf*. The sequence −244 to +1 bp relative to the P*sufA* transcription start site (TSS) was replaced with *kan-P_fnr_-suf_EC_* (containing −155 to +1 bp relative to the *P_fnr_* TSS from pPK6980) by PCR amplification and electroporation into MG1655 containing pKD46 to generate PK16191. The *kan-P_fnr_-suf_EC_* allele was then P1 transduced to PK14464 (MG1655 Δ*lacY*Δ*ispH::cat putP::*Δ*kan* P*_BAD_* MVA*^+^*Δ*lacZYA::tet*), resulting in PK14842. Replacement of the *E. coli suf* operon in PK16191 with the *Z. mobilis suf* operon and *sufE* occurred in several steps. First, Gibson assembly and PCR amplification were used to construct a plasmid in which *kan*-P*_fnr_* was driving expression of the *Z. mobilis sufBCDSTAE* (*suf_ZM_*). The resulting *kan-P_fnr_-suf_ZM_* allele was then PCR-amplified and electroporated into MG1655 Δ*sufABCDSE_EC_*::FRT-*cat*-FRT P*_BAD_- sufBCDSTAE_ZM_* containing pKD46, such that *kan-P_fnr_-suf_ZM_* replaced FRT-*cat*-FRT P*_BAD_* to yield MG1655 *kan-P_fnr_-suf_ZM_. kan*-P*_fnr_-suf_ZM_* was P1 transduced to PK14464, resulting in PK14862. Plasmids carrying the *E. coli* or *Z. mobilis ispH* or *Z. mobilis ispHispG* were transformed into PK14862.

### Complementation of *E. coli* Δ*ispH*, Δ*ispG,* and Δ*ispH*Δ*ispG* mutant strains

*E. coli* strains with relevant plasmids were tested for their ability to complement the indicated mutations by growth in the absence of mevalonate under aerobic and anaerobic conditions. Strains were initially grown overnight in LB media containing arabinose (10 mM), mevalonate (1.0 mM), and spectinomycin (100 μg/mL). The cell suspension was normalized to optical density at 600 nm (OD_600_) of 1.0, and 1.0 mL was centrifuged for 2 min, the supernatant was discarded, and the pellet was washed once with LB to remove mevalonate. The cells were pelleted again, resuspended in 1 mL of LB, and 5 μL of serial dilutions was spotted on tryptone yeast extract (TYE) agar plates containing either mevalonate (1 mM), arabinose (10 mM), IPTG (0, 25 μM, 50 μM, 100 μM, or 200 μM), and spectinomycin (100 μg/mL) or TYE agar plates containing only IPTG (0, 25 μM, 50 μM, 100 μM, or 200 μM) and spectinomycin (100 μg/mL). Plates were incubated in air for aerobic growth or in a sealed jar with AnaeroPack System (Mitsubishi Gas Chemical) for anaerobic growth at 37°C for 16 hours. Each strain was assayed at least three times.

### Western blot assay

Levels of plasmid-encoded C-terminal Strep-tag II IspG or Strep-tag II IspH produced following IPTG induction were measured in relevant *E. coli* strains by western blotting. Strains were grown overnight in LB media containing mevalonate (1 mM), arabinose (10 mM), and spectinomycin (100 μg/mL) for 16 hours at 37°C. Bacterial strains were sub-cultured (1:100) into the same media until the OD_600_ reached 0.2, and then, protein expression was induced by the addition of IPTG (25 μM, 50 μM, 100 μM, or 200 μM) at the final concentration. After 2 hours, the cells were normalized to OD_600_ of 0.2, and 1 mL of cell suspension was pelleted. Cell pellets were resuspended in 125 μL of 1× sodium dodecyl-sulfate (SDS)-loading buffer, heated to 95°C for 5 min, and 10 μL (2 × 10^5^ cells) of the sample was electrophoresed by sodium dodecyl-sulfate polyacrylamide gel electrophoresis (SDS-PAGE). The gel was transferred to a 0.45 µm nitrocellulose membrane (Amersham Protran), and the assay was performed with Strep-tag II antibody horseradish peroxidase (HRP) conjugate (EMD Millipore) according to the manufacturer’s recommendations. The blot was imaged using an Azure imager and was analyzed using AzureSpot Pro software. Protein levels were measured from three biological replicates for each strain, induced at four concentrations of IPTG.

### Expression and purification of *Z. mobilis* IspH, *Z. mobilis* IspG, and *E.coli* IspH

PK13681 that contains the repaired *suf* operon (32) controlled by arabinose-induced P*_BAD_* promoter (23) was transformed with pET11a derivatives containing either *Z. mobilis* IspH with a Strep-tag II at the C-terminus (pPK13650), *Z. mobilis* IspG with a Strep-tag II at the C-terminus (pPK13651), or *E.coli* IspH with a Strep-tag II at the C-terminus (pPK13648). Cultures were grown aerobically at 37°C in 1 L of terrific broth (Research Products International) containing final concentrations of 10 mg/L ferric ammonium citrate, 2 mM cysteine, 10 mM arabinose, and 100 μg/ mL ampicillin. At an OD_600_ of 0.3, synthesis of strep-tagged protein was induced by adding IPTG to a final concentration of 0.4 mM for 2 hours. The induced culture was sparged overnight (20 hours) with argon at 4°C. The culture was centrifuged at 8,000 rpm in a Beckman JLA 10.500 rotor for 15 min at 4°C, resuspended in 50 mM Tris-HCl (pH 8), 150 mM NaCl, 1 mM dithiothreitol (DTT), and 100 μM phenylmethylsulfonyl fluoride (PMSF) under anaerobic conditions and lysed in a French press at 20,000 psi. The lysate was centrifuged at 45,000 rpm in a Beckman 70.1 Ti rotor for 1 hour at 4°C. The supernatant was fractionated using an AKTA pure chromatography system (Cytiva) equipped with a Streptrap HP column (5 mL, GE Healthcare), equilibrated with 50 mM Tris-HCl (pH 8), 150 mM NaCl, and 1 mM DTT. Strep-tagged proteins were eluted with 50 mM Tris-HCl (pH 8), 150 mM NaCl, 2.5 mM desthiobiotin, and 1 mM DTT. The DTT and desthiobiotin in the pooled protein fraction were removed using a PD-10 desalting column containing 8.3 mL of Sephadex G-25 resin (Cytiva) with 50 mM Tris-HCl (pH 7.5), 150 mM NaCl. The concentration of the protein was measured using Bradford assay (33), and iron was measured with tripyridyl-S-triazine (34). The [4Fe-4S] cluster occupancy ranged between 5% and 10%. The isolation of proteins was carried out anaerobically inside a Coy anaerobic chamber with an atmosphere of 90% N_2_ and 10% H_2_.

### [4Fe-4S] cluster stability of IspH and IspG

Anaerobically isolated *Z. mobilis* Strep-tag II IspG (60 μM), *Z. mobilis* Strep-tag II IspH (60 μM), or *E. coli* Strep-tag II IspH (60 μM) in 50 mM Tris-HCl (pH 7.5) and 150 mM NaCl was placed in sealed cuvettes in an anaerobic chamber. After collection of the anaerobic UV-visible spectrum in a Perkin Elmer Lambda 25 spectrophotometer, the cuvettes were opened to air, and the absorbance spectra were monitored at 30-min intervals. The absorbance at 410 nm from these spectra was plotted versus time as a measure of [4Fe-4S] cluster stability.

### Bacterial two-hybrid assay

BTH101 was transformed with pKT25-zip and pUT18C-zip, pKT25 and pUT18C, pKT25-*ispH_EC_* and pUT18C-*ispG_EC_*, pKT25-*ispG*_*EC*_ and pUT18C-*ispG*_*EC*_, and pKT25-*ispH*_*ZM*_ and pUT18C-*ispG*_*ZM*_. *Z. mobilis* IspH, *E. coli* IspH, or *E. coli* IspG were fused in frame downstream of the T25 domain encoded on plasmid pKT25 at the KpnI site. *Z. mobilis* IspG or *E. coli* IspG were fused in frame downstream of T18 on plasmid pUT18C at the KpnI site. The plasmids were assembled by PCR using the primers indicated in Table S1 and Gibson assembly. The transformants were screened on X-gal (40 μg/mL, IPTG (500 μM), ampicillin (200 μg/mL), and kanamycin (50 μg/mL) under aerobic and anaerobic conditions.

### Molecular sieve chromatography

A Superdex 200 Increase 10/300 Gl column equilibrated in 50 mM Tris-HCl (pH 7.5) and 150 mM NaCl connected to a AKTA FPLC (Cytiva) was calibrated with Biorad gel filtration standards (thyroglobulin, gamma globulin, ovalbumin, myoglobulin, and vitamin B12). *Z. mobilis* Strep-tag-II-IspG (50 μM) or Strep-tag-II-IspH (50 μM) or an equimolar mixture of both (25 μM each) was loaded on the column under anaerobic conditions, and the elution profile was monitored by the absorbance at 280 nm.

### Native-PAGE gel

Standard molecular weight markers (ThermoFisher), 50 μM IspG, 50 μM IspH, and a mixture of 25 μM each of IspH and IspG were electrophoresed on a 4%–16% Bis-Tris Native gel (ThermoFisher) according to the manufacturer’s recommendations.

## RESULTS

### *Z. mobilis* IspG and IspH partially complement an *E. coli* mutant lacking the analogous proteins under anaerobic conditions

**Fig 1:**
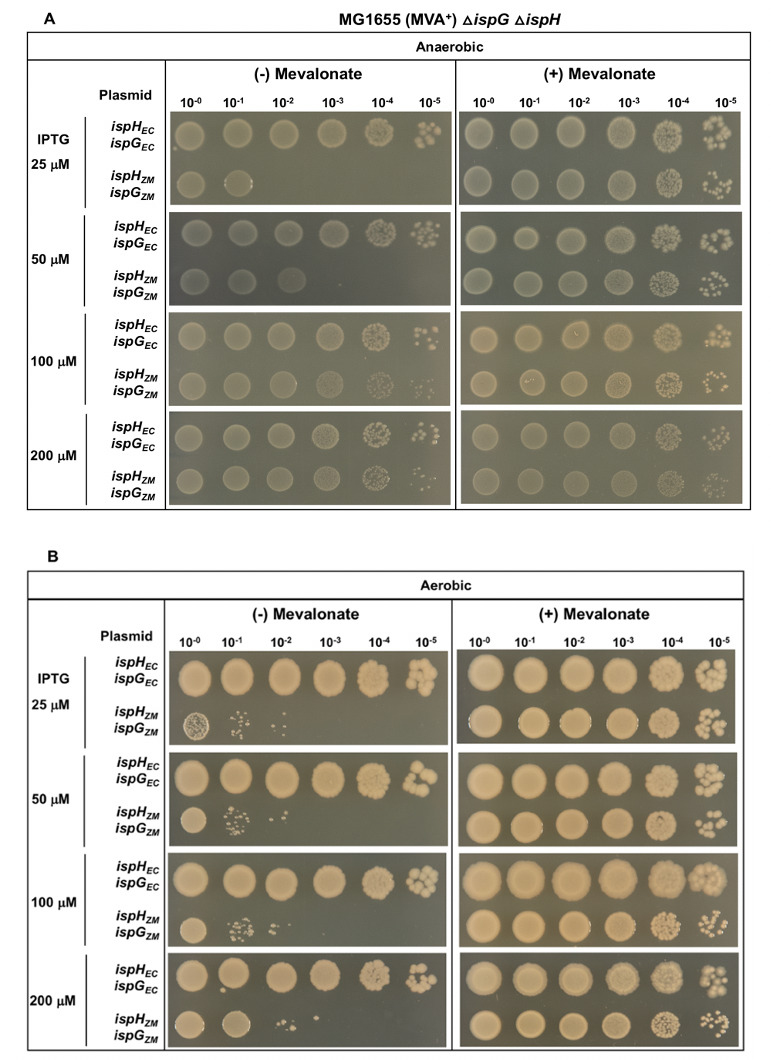
*E. coli*Δ*ispG*Δ*ispH* MVA^+^ (PK14487) with plasmid variants containing *ispG* and *ispH* from either *Z. mobilis* (*ispH_ZM_ ispG_ZM_*) or *E. coli* (*ispH_EC_ ispG_EC_*) were grown in LB with mevalonate, arabinose, and spectinomycin. After 16 hours of overnight growth, bacteria were washed with LB to remove mevalonate and were normalized to OD_600_ of 1. The bacteria were then serially diluted in LB, and 5 μL was used from each dilution tube to spot on solid TYE media, with or without mevalonate (1.0 mM), containing the indicated concentrations of IPTG (25 μM, 50 μM, 100 μM, or 200 μM) to induce *ispG* and *ispH* co-expression. The agar plates were then incubated at 37°C overnight under either (A) anaerobic or (B) aerobic conditions.

The function of IspG and IspH from *Z. mobilis* was compared with analogous proteins from *E. coli* using an *E. coli* strain deleted for chromosomal *ispG* and *ispH,* but containing a gene cassette that maintains viability by conditional production of isoprenoids from the eukaryotic mevalonic acid (MVA) pathway when supplied with mevalonate. As indicated in Table 1 and Methods, the genotype of this base strain is MG1655 Δ*lacY putP::*Δ *kan P_BAD_ yPMD, hPMK, yMVK, ecIDI*, Δ*lacZYA::tet* but is abbreviated as *E. coli* MVA^+^ here and in the figure legends. Expression of plasmid-encoded *ispG* and *ispH* for comparison of *Z. mobilis* to *E. coli* proteins was controlled by an IPTG-inducible promoter. Complementation of *E. coli*Δ*ispG*Δ*ispH* MVA^+^ (PK14487) with indicated plasmids was assayed by plating serial dilutions of liquid cultures grown under permissive conditions (with mevalonate) onto solid media with or without mevalonate under anaerobic conditions at the indicated IPTG concentrations (Fig. 1A). Plasmid-encoded *E. coli ispG* and *ispH* fully rescued the growth of this strain over a range of IPTG concentrations (0, 25 μM, 50 μM, 100 μM, and 200 μM) since the number and size of the colonies were similar at the highest dilution tested (10^−5^) regardless of whether mevalonate was present. Indeed, even in the absence of IPTG, sufficient IspH and IspG are produced to complement the mutant (Fig. S1A). In contrast, plasmid-encoded *Z. mobilis ispG* and *ispH* were unable to fully complement the mutant phenotype at 0, 25, and 50 μM IPTG as growth was not observed beyond the 10^−2^ dilution in the absence of mevalonate, in contrast to the robust growth observed with mevalonate. Growth at the 10^−1^ dilution is the sensitivity limit of this assay and likely represents a dilution of the preexisting pool of mevalonate and mevalonate pathway components since we observe some growth up to the 10^−1^ dilution with the plasmid vector alone (Fig. S1B). At 100 and 200 μM IPTG induction, colonies were observed with *Z. mobilis ispG* and *ispH* at the 10^−5^ dilution similar to *E. coli* (Fig. 1A). However, the size of the colonies was smaller in the absence of mevalonate than with mevalonate and smaller than those produced by *E. coli ispG* and *ispH*. These results indicate that *Z. mobilis* IspG and IspH function to produce isoprenoid precursors in *E. coli*, although less efficiently than their *E. coli* counterparts.

### *Z. mobilis* IspG is not as functional as *E. coli* IspG under anaerobic conditions

**Fig 2:**
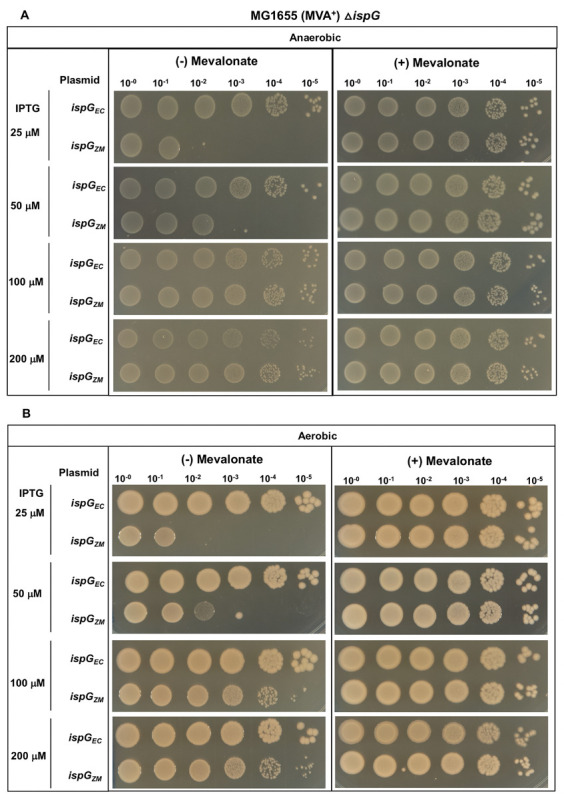
*E. coli*Δ*ispG* MVA^+^ (PK14465) with plasmid variants containing *ispG* from *Z. mobilis* (*ispG_ZM_*) or *E. coli* (*ispG_EC_*) were grown in LB with mevalonate, arabinose, and spectinomycin. Viability of cells was assayed as described in Fig. 1.

To dissect whether both *Z. mobilis ispG* and *ispH* were equally inefficient in producing isoprenoids in *E. coli*, we tested the function of each gene separately. Using *E. coli*Δ*ispG* MVA^+^ (PK14465), we compared the viability of strains containing plasmid-encoded *E. coli* and *Z. mobilis ispG* using the same growth assay. As expected, plasmid-encoded *E. coli ispG* was able to restore growth at all IPTG concentrations as colonies were observed at each 10^−5^ dilution. In contrast, complementation by *Z. mobilis ispG* was inefficient at lower IPTG concentrations (25 and 50 μM IPTG) but was similar to *E. coli ispG* at 100 and 200 μM IPTG, and the colonies observed at the 10^−5^ dilutions were similar in size to that of the permissive condition (Fig. 2A). Overall, the pattern of results mostly mirrored the complementation of Δ*ispG*Δ*ispH* strain by *Z. mobilis ispG* and *ispH*. Western blot analysis of Strep-tag II IspG demonstrated that protein levels of *E. coli* and *Z. mobilis* IspG were similar following liquid growth at the various IPTG concentrations (Fig. S3 and S4). Thus, it is likely that differences in activity and not protein levels explain the partial complementation by *Z. mobilis* IspG.

### *Z. mobilis* IspH is less functional than *Z mobilis* IspG in complementing their respective *E. coli* mutants under anaerobic conditions

**Fig 3:**
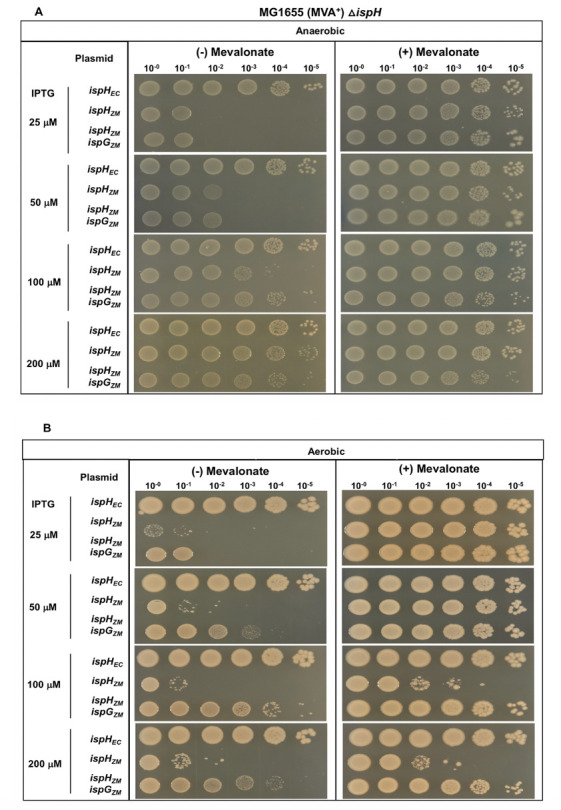
*E. coli*Δ*ispH* MVA^+^ (PK14464) with plasmid variants containing *ispH* from *Z. mobilis* (*ispH_ZM_*) or *E. coli* (*ispH_EC_*) or *ispG* and *ispH* from *Z. mobilis* (*ispH_ZM_ ispG_ZM_*) were grown in LB with mevalonate, arabinose, and spectinomycin. Viability of cells was assayed as described in Fig. 1.

The function of *Z. mobilis ispH* was compared with that of its *E. coli* ortholog using the growth of *E. coli*Δ*ispH* MVA^+^ (PK14464) as an assay. As expected, plasmid-encoded *E. coli ispH* was able to complement the function of the mutant at all IPTG concentrations. In contrast, *Z. mobilis ispH* only partially complemented *E. coli*Δ*ispH* MVA^+^ at 50 and 100 μM IPTG, and no complementation was observed at 25 μM IPTG. However, at 200 μM IPTG, a similar number of colonies was present as found with *E. coli ispH,* although the colony size was smaller compared with *E. coli ispH* or when grown on media containing mevalonate (Fig. 3A). Western blot analysis of Strep-tag II IspH demonstrated that protein levels of *E. coli* and *Z. mobilis* IspH were similar following liquid growth at the various IPTG concentrations assayed (Fig. S4). Thus, it is likely that differences in activity and not protein levels explain the difference in complementation between *Z. mobilis* and *E. coli* IspH. Taken together, *Z. mobilis ispH* was less efficient than *Z. mobilis ispG* in complementing the corresponding *E. coli* mutants and less efficient than *Z. mobilis ispG* and *ispH* in complementing Δ*ispG*Δ*ispH,* suggesting a requirement for its cognate partner, IspG.

### *Z. mobilis* IspH function is optimal when paired with its cognate IspG

**Fig 4:**
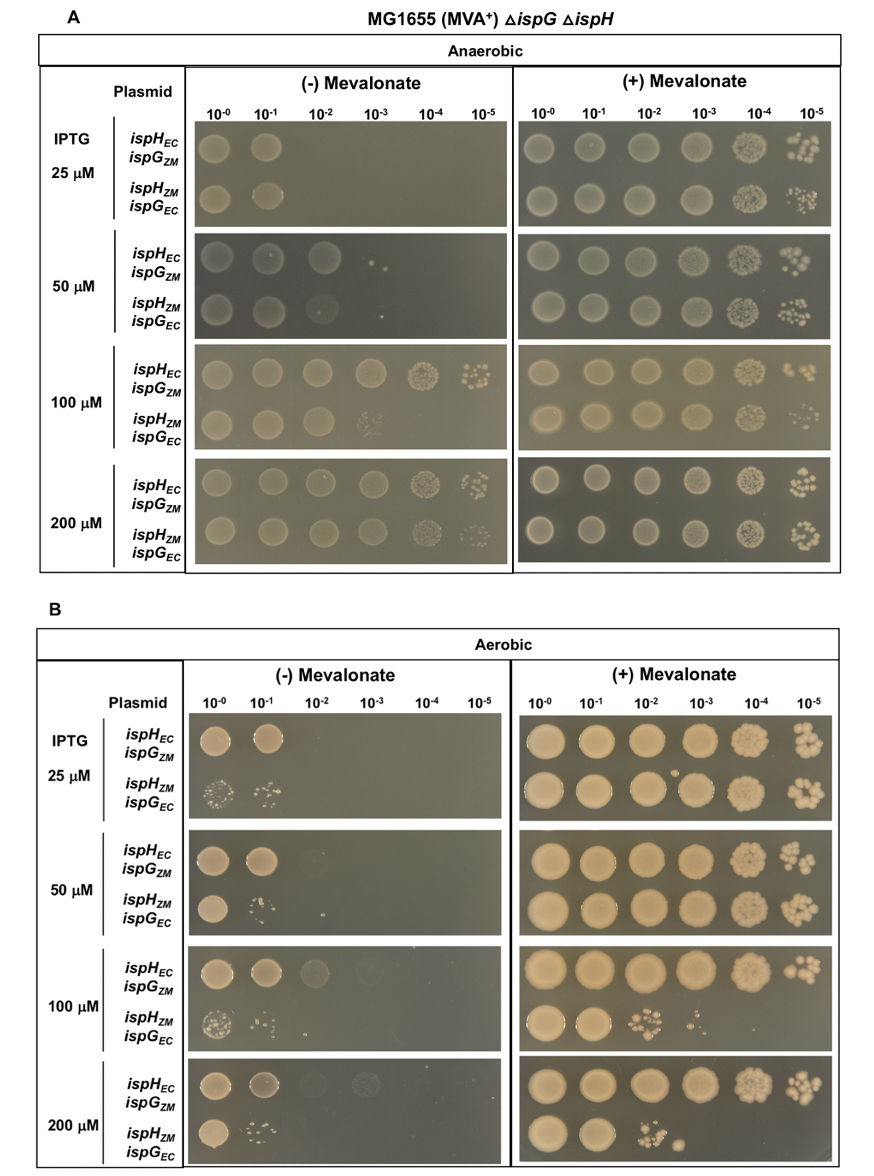
*E. coli*Δ*ispG*Δ*ispH* MVA^+^ (PK14487) with plasmid variants containing *ispG* from *E. coli* and *ispH* from *Z. mobilis* (*ispH_ZM_ ispG_EC_*) or *ispG* from *Z. mobilis* and *ispH* from *E. coli* (*ispH_EC_ ispG_ZM_*) were grown in LB with mevalonate, arabinose, and spectinomycin. Viability of cells was assayed as described in Fig. 1.

To test the preference of IspG and IspH for their cognate partner, the analogous genes from each species were swapped. When *E. coli ispG* was co-expressed with *Z. mobilis ispH* in *E. coli*Δ*ispG*Δ*ispH* MVA^+^ (PK14487), we observed that this pair functioned more poorly in complementation under anaerobic conditions (Fig. 4A) than either *Z. mobilis ispG* and *ispH* or *E. coli ispG* and *ispH* (Fig. 1A), indicating that *E. coli* IspG was not an adequate substitute for *Z. mobilis* IspG. In addition, when *E. coli ispH* was co-expressed with *Z. mobilis ispG*, we observed that the complementation efficiency was reduced (Fig. 4A) compared with *E. coli ispG* and *ispH* and slightly increased compared with *Z. mobilis ispG* and *ispH* (Fig. 1A), suggesting that *E. coli* IspH could function with *Z. mobilis* IspG but less efficiently than *E. coli* IspG. These data indicate that IspH function exhibits a preference for its cognate IspG, suggesting that *ispG* and *ispH* may have co-evolved to function optimally with its cognate partner through an unknown mechanism.

We next tested if co-expressing *Z. mobilis ispG* with *ispH* improved the function of *Z. mobilis* IspH under anaerobic conditions in PK14463 lacking *E. coli ispH* but retaining chromosomal *E. coli ispG*. We observed a 10-fold increase in the number of colonies at 100 μM IPTG with plasmid-encoded *Z. mobilis ispG ispH* than with *ispH* alone (Fig. 3A), indicating that co-expression of *Z. mobilis* IspH with IspG improved complementation of *E. coli*Δ*ispH* MVA^+^ (PK14464). To address whether co-expression with IspG altered IspH protein levels, western blot analysis of Strep-tag II IspH was performed from cultures grown with a range of IPTG concentrations, which showed that levels of *Z. mobilis* IspH did not change significantly in the presence or absence of IspG (Fig. S4). Altogether, our data indicate that *Z. mobilis* IspH functions best in this assay with IspG from the same organism.

### *Z. mobilis* IspG and IspH confer an O_2_-sensitive phenotype to *E. coli*Δ*ispG*Δ*ispH*

IspG and IspH each have [4Fe-4S] cluster cofactors that are generally considered O_2_ sensitive (20), and in *Z. mobilis,* the substrates of these enzymes accumulate when anaerobic cultures are shifted to aerobic conditions (9). To determine if there were differences in enzyme function in the presence of O_2_ when the clusters were expected to be less stable, we used the same complementation assay described above but incubated plates under aerobic conditions. Plasmid-encoded *E. coli ispG* and *ispH* complemented the function of *E. coli*Δ*ispG*Δ*ispH* MVA^+^ (PK14487) at all IPTG concentrations (25–200 μM) similar to anaerobic conditions. In contrast, *Z. mobilis ispG* and *ispH* showed at least a 100-fold reduction in viability under aerobic conditions (Fig. 1B) compared with anaerobic conditions at 100 and 200 μM IPTG (Fig. 1A). This result suggests that the *Z. mobilis* IspG and IspH [4Fe-4S] clusters may be more sensitive to O_2_ than their *E. coli* orthologs, conferring an O_2_-sensitive phenotype to growth.

### *Z. mobilis* IspG does not confer O_2_ sensitivity to *E. coli*

To examine whether both *Z. mobilis ispG* and *ispH* were responsible for the O_2_-sensitive phenotype in *E. coli*, we tested the function of each gene individually under aerobic conditions. As expected, plasmid-encoded *E. coli ispG* was able to restore the growth of *E. coli*Δ*ispG* MVA^+^ (PK14465) at all IPTG concentrations. For *Z. mobilis ispG,* the pattern of growth observed under aerobic conditions (Fig. 2B) was nearly identical to that observed under anaerobic conditions (Fig. 2A); there appears to be only a small reduction in colony forming units at equivalent IPTG concentrations under aerobic conditions. These results suggest that *Z. mobilis* IspG is not the major driver of the O_2_-sensitive phenotype observed with both *Z. mobilis* IspG and IspH.

### *Z. mobilis* IspH confers an O_2_-sensitive phenotype to *E. coli*

Using *E. coli*Δ*ispH* MVA^+^ (PK14464), we compared the function of *E. coli* and *Z. mobilis ispH* under aerobic conditions. As expected, plasmid-encoded *E. coli ispH* was able to fully complement the function of *E.coli*Δ*ispH* MVA^+^ under aerobic conditions. In contrast, *Z. mobilis ispH* was severely defective in complementation at all IPTG concentrations (Fig. 3B) compared with anaerobic conditions (Fig. 3A). This phenotype was not due to differences in protein levels as determined by western blots of cells grown under anaerobic (Fig. S3 and S4) and aerobic conditions (Fig. S3 and S5).

### The [4Fe-4S] clusters of IspG and IspH are sensitive to O_2_ *in vitro*

**Fig 5:**
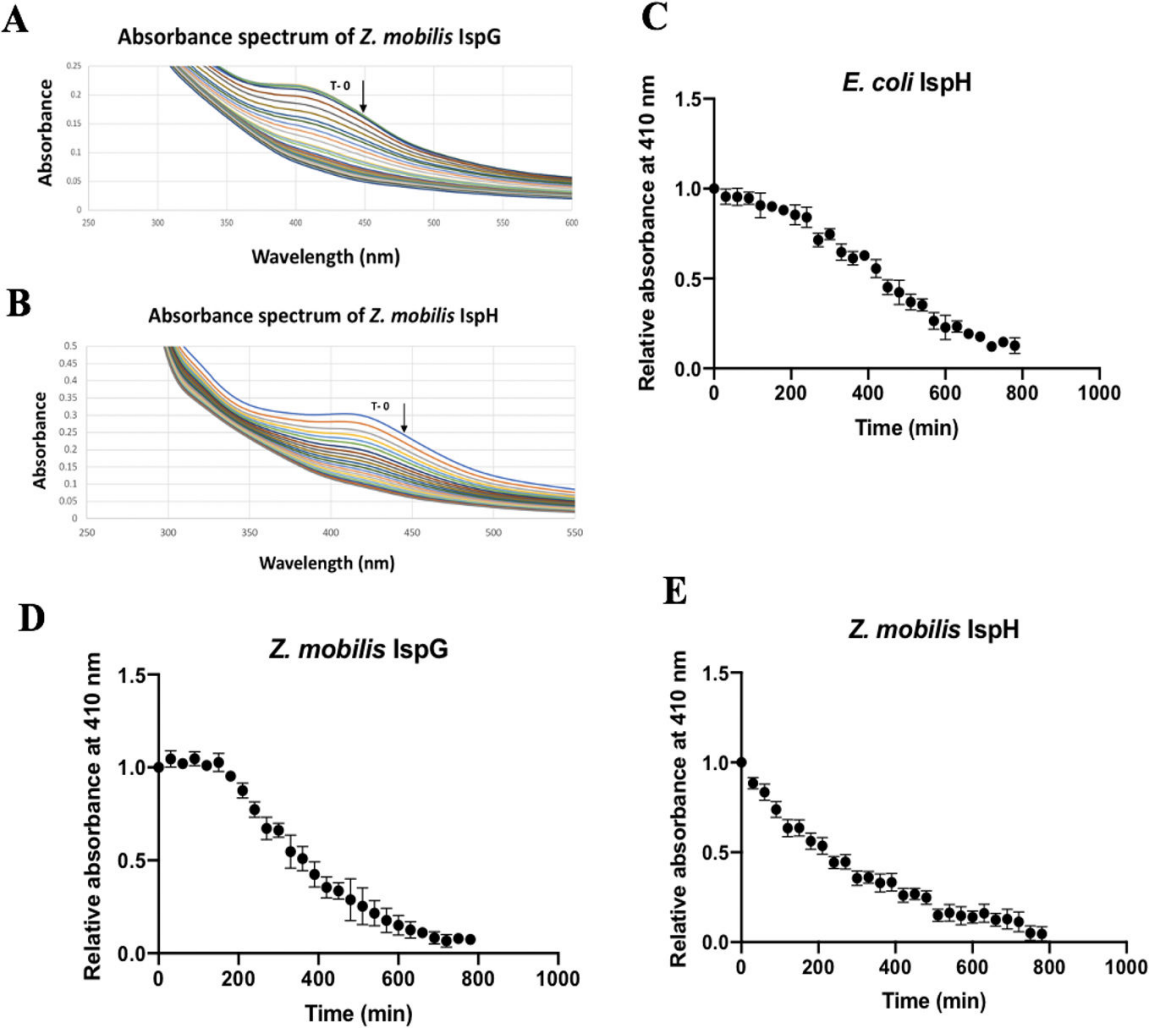
Loss of the [4Fe-4S] cluster from IspG and IspH following exposure to air. UV-visible absorbance changes of anaerobically isolated *Z. mobilis* Strep-tag-II-IspG (60 μM) (**A**) and *Z. mobilis* Strep-tag-II-IspH (60 μM) (**B**) following exposure to air. (**C**) Plot of the change in absorbance at 410 nm after exposure of *E. coli* Strep-tag II IspH to air. (**D**) Plot of the change in absorbance at 410 nm from the data in panel A. (**E**) Plot of the change in absorbance at 410 nm from the data in panel B. Relative absorbance at 410 nm is the normalized absorbance value at the initial time point.

Since aerobic conditions impaired the function of *Z. mobilis* IspH in the complementation assays, we tested the stability of the [4Fe-4S] cluster of *Z. mobilis* IspG, *Z. mobilis* IspH, and *E. coli* IspH to air *in vitro* (Fig. 5). The proteins were isolated anaerobically via a Strep-Tag II at their C-termini, yielding protein with the characteristic absorbance spectra of a [4Fe-4S] cluster (Fig. 5A and B). Anaerobic protein (60 μM) was then exposed to air, and the change in the characteristic absorbance peak at 410 nm was monitored over a 12-hour time period. We used *E. coli* IspH as a control since the lability of the [4Fe-4S] cluster to O_2_ was previously reported (20). All three proteins lost the [4Fe-4S] cluster absorbance over time, indicating their sensitivity to O_2_ (Fig. 5C through E). Although fitting the data with GraphPad Prism revealed a shorter half-life of the [4Fe-4S] cluster of *Z. mobilis* IspH (237 min) compared with *Z. mobilis* IspG (349 min) and *E.coli* IspH (496 min), differences in the initial cluster occupancy between the three samples precluded a more rigorous analysis.

### *Z. mobilis* IspH catalytic activity is required to impart an O_2_-sensitive growth defect with mevalonate

**Fig 6:**
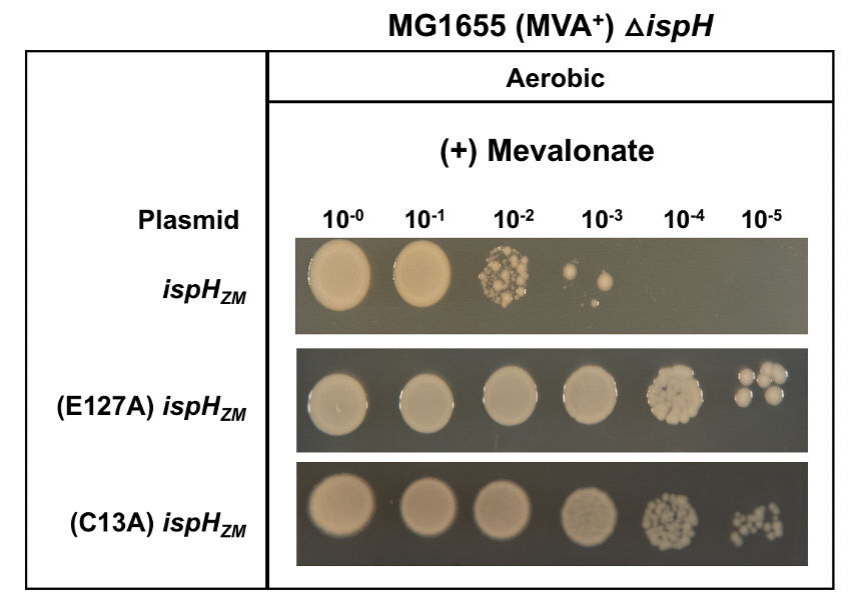
*E. coli*Δ*ispH* MVA^+^ (PK14464) with plasmid variants containing *ispH* from *Z. mobilis* (*ispH_ZM_*) with point mutations E127A or C13A were grown in LB with mevalonate, arabinose, and spectinomycin. Viability of cells was assayed as described in Fig. 1 at 100 μM IPTG.

Unexpectedly, an O_2_-sensitive phenotype was also observed for the expression of *Z. mobilis ispH* in *E. coli*Δ*ispH* MVA^+^ (PK14464) in the presence of mevalonate as colonies were only observed at 10^−3^ dilution at 100 and 200 μM IPTG (Fig. 3B), suggesting a phenotype that could not be explained simply by the lack of isoprenoids. We considered the possibility that *Z. mobilis* IspH could be producing an off-pathway inhibitory product under aerobic conditions since promiscuous activities of other IspHs have been noted previously (18, 19). Thus, we tested whether the O_2_-sensitive phenotype in the presence of mevalonate depended on the catalytic function of *Z. mobilis* IspH. Substitution of *Z. mobilis* IspH Glu127 to Ala should inactivate enzyme activity based on the analogous *E. coli* active site mutant, E126A (16). In addition, we constructed a second substitution, *Z. mobilis* IspH C13A that should also inactivate enzyme activity because it removes one of the Cys expected to ligate the [4Fe-4S] cluster. Both *Z. mobilis* IspH variants restored aerobic growth to mevalonate-grown cells as colonies were observed at 10^−5^ dilution (Fig. 6). Furthermore, expression of *Z. mobilis ispH* in a strain containing a wild-type copy of *E. coli ispH* does not have an oxygen-sensitive growth phenotype, indicating that function of *E. coli* IspH suppresses this activity (Fig. S6). These results suggest that *Z. mobilis* IspH has an aberrant activity in the presence of O_2_.

### The cognate-enzyme pair is required to suppress the aberrant O_2_-dependent activity of *Z*. *mobilis* IspH

We reasoned that expression of *Z. mobilis* IspG might be necessary to eliminate this aberrant activity because growth inhibition with mevalonate was not observed when complementing *E. coli* Δ*ispG*Δ*ispH* MVA^+^ with *Z. mobilis ispG* and *ispH*. Indeed, co-expression of *Z. mobilis ispG* with *Z. mobilis ispH* significantly improved the growth of MG1655Δ*ispH* MVA^+^ in both the presence and absence of mevalonate at 100 and 200 μM IPTG (Fig. 3B). This improvement in growth suggests that *Z. mobilis* IspG prevents this aberrant activity by *Z. mobilis* IspH by an unknown mechanism.

The preference of *Z. mobilis* IspH for *Z. mobilis* IspG was additionally demonstrated in *E. coli*Δ*ispG*Δ*ispH* MVA^+^ (PK14487) in a swapping experiment with *E. coli* IspG (Fig. 4A and B). When *E. coli ispG* was co-expressed with *Z. mobilis ispH,* the same O_2_-sensitive phenotype in the presence of mevalonate was observed. In addition, in the absence of mevalonate, a nearly 100-fold reduction in viability was observed with 100 and 200 μM IPTG under aerobic conditions compared with growth under anaerobic conditions. Interestingly, *E. coli* IspH does not show the O_2_-dependent aberrant activity when it is co-expressed with heterologous IspG from *Z. mobilis* since there is no loss in viability in the presence of mevalonate under aerobic conditions. However, we did observe that in the absence of mevalonate, co-expression of *Z. mobilis ispG* with *E. coli ispH* impedes complementation of *E. coli*Δ*ispG*Δ*ispH* under aerobic conditions, although we did not observe a similar effect when complementing *E. coli*Δ*ispG* with *Z. mobilis ispG* under aerobic or anaerobic conditions. Since the presumed difference between these two strains is the level of *E. coli* IspH, it suggests that *E. coli* IspH might compete with *Z. mobilis* IspG for a common cofactor under aerobic conditions impeding the activity of *Z. mobilis* IspG. Since one common cofactor is the [4Fe-4S] cluster, we investigated if the Fe-S cluster biogenesis machinery was limiting under aerobic conditions.

### Expression of the *Z. mobilis suf* operon in *E. coli* does not improve the function of *Z. mobilis* IspG or IspH

**Fig 7:**
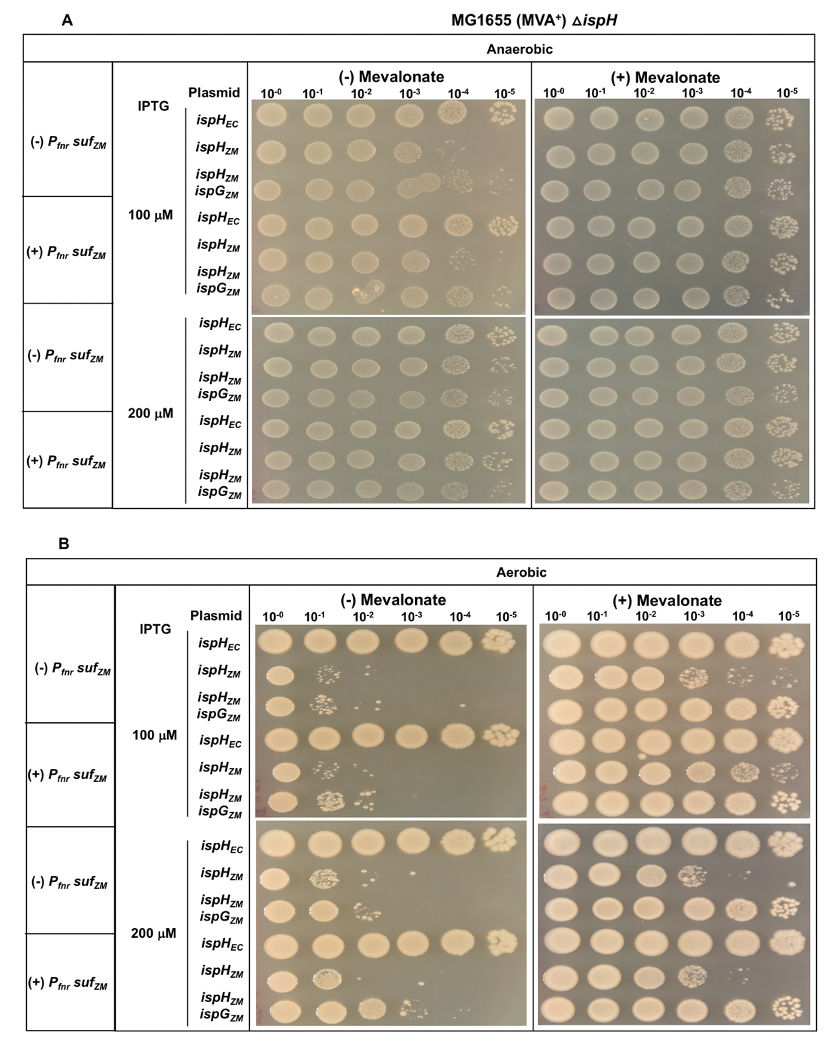
*E. coli*Δ*ispH* MVA^+^ or *E. coli*Δ*ispH P_fnr_ suf_ZM_* MVA^+^ with plasmid variants containing *ispH* from *Z. mobilis* (*ispH_ZM_*) or *E. coli* (*ispH_EC_*) or *ispG* and *ispH* from *Z. mobilis* (*ispH_ZM_ ispG_ZM_*) were grown in LB with mevalonate, arabinose, and spectinomycin. Viability of cells was assayed as described in Fig. 1.

**Fig 8:**
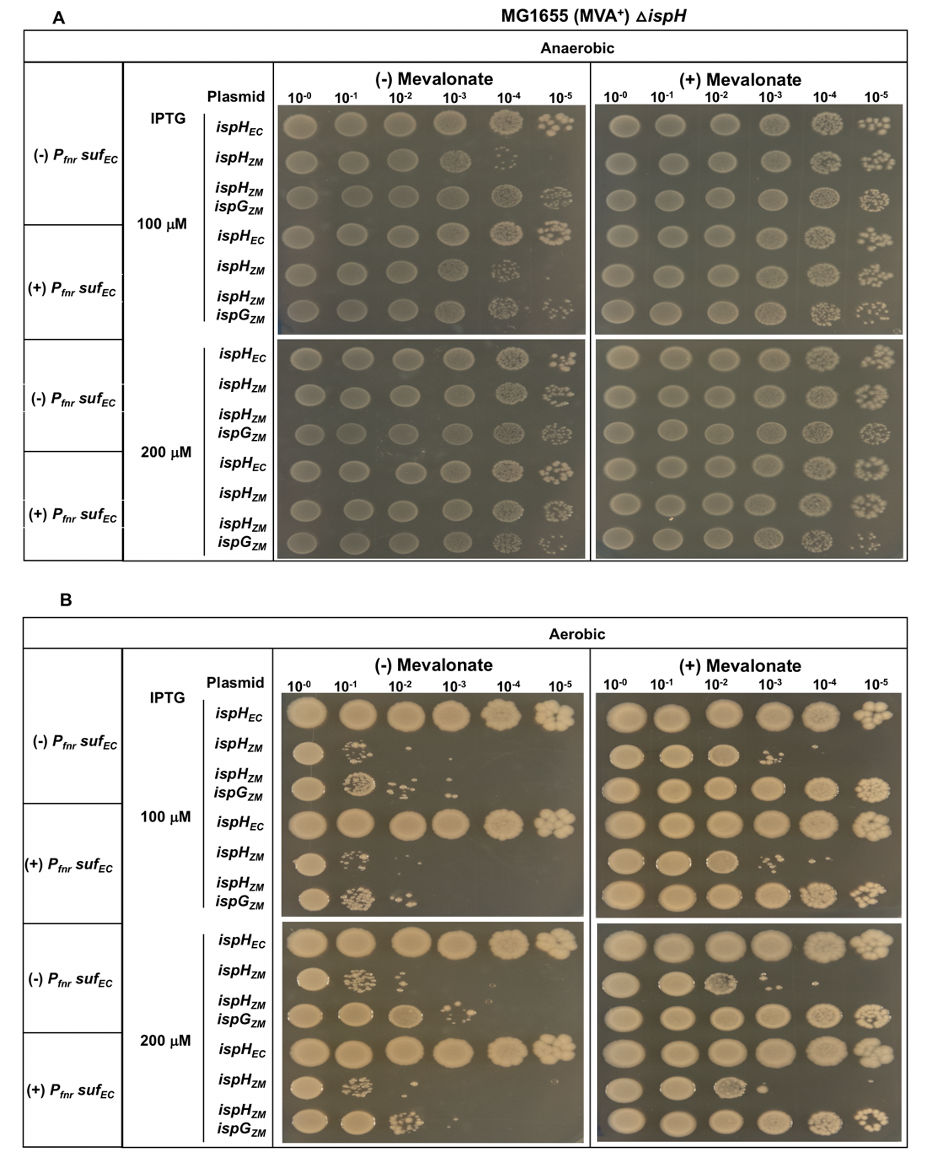
*E. coli*Δ*ispH* MVA^+^ or *E. coli*Δ*ispH P_fnr_ suf_EC_* MVA^+^ with plasmid variants containing *ispH* from *Z. mobilis* (*ispH_ZM_*) or *E. coli* (*ispH_EC_*) or *ispG* & *ispH* from *Z. mobilis* (*ispH_ZM_ ispG_ZM_*) were grown in LB with mevalonate, arabinose, and spectinomycin. Viability of cells was assayed as described in Fig. 1

We focused our attention on the SUF Fe-S cluster biogenesis pathway since *Z. mobilis* has only the SUF pathway for producing its complement of Fe-S proteins, whereas *E. coli* has both the ISC and SUF pathways. In *E. coli*, the housekeeping ISC pathway was previously shown to maturate *E. coli* IspG and IspH. To test whether the inefficient complementation of *Z. mobilis* IspG and IspH resulted from insufficient Fe-S cluster maturation due to differences in biogenesis pathways, *E. coli* strains were constructed that had the native *E. coli suf* operon replaced with that from *Z. mobilis* and placed under control of a constitutive promoter. Under either aerobic or anaerobic conditions, we observed no improvement in the complementation of *E. coli*Δ*ispH* MVA^+^ with the *Z. mobilis* SUF machinery for either expression of *Z. mobilis ispH* or Z. mobilis *ispH* and *ispG* at 100–200 μM IPTG (Fig. 7). As a control, we tested the native *E. coli suf* operon expressed from the same constitutive promoter, and it also showed no difference in complementation efficiency (Fig. 8). Western blot analysis confirmed the higher level of SufD in this strain compared with the native promoter (Fig. S7).

### Does IspG physically interact with IspH?

As the presence of IspG improved the function of IspH from *Z. mobilis*, we also considered the possibility of whether IspG and IspH form a specific complex. Using two *in vitro* assays to test for physical association (molecular sieve chromatography and native gels), we did not observe complex formation between *Z. mobilis* IspG and IspH under the conditions tested. For molecular sieve chromatography, anaerobically isolated Strep-tag-II IspH (25 μM) and IspG (25 μM) were mixed together and loaded onto a Superdex 200 Increase 10/300 Gl molecular sieve column under anaerobic conditions (Fig. S8A). The elution profile of this equimolar mixture reflected the sum of the individual profiles of IspG or IspH, indicating no physical interaction could be detected under these solution conditions. Native polyacrylamide gel electrophoresis analysis of an equimolar mixture of *Z. mobilis* IspG (25 μM) and IspH (25 μM) also showed that the proteins separated according to their native size, which for IspG was a dimer (79.8 kDa) and for IspH was a monomer (37 kDa) (Fig. S8B).

Since the reactions catalyzed by these proteins also require partner proteins, such as ferredoxins or flavodoxins, we reasoned that a cell-based assay might detect an interaction if other proteins were also required. We used the bacterial adenylate cyclase two*-*hybrid (BACTH) assay (25) for this analysis where fusion proteins to two different domains of adenylate cyclase will reconstitute activity and produce cAMP if the tested proteins oligomerize. Activity is assayed in *E. coli*Δ*cya* through the CAP-cAMP-dependent expression of the *lac* operon. IspG from *E. coli* and *Z. mobilis* were individually fused with T18 at their N-termini, and IspH from *Z. mobilis* and *E. coli* were individually fused with T25 at their N-termini, engineered on pUT18C and pKT25, respectively. Restoration of adenylate cyclase activity under aerobic and anaerobic conditions was assayed by plating transformed strains on TYE X-gal, IPTG (500 μM), ampicillin (200 μg/mL), and Kan (50 μg/mL). Blue colonies were detected with the positive control, pKT25-zip and pUT18C-zip, containing strain (Fig. S8C). However, for the combinations of *Z. mobilis* IspG and IspH or *E. coli* IspH and IspG tested, we observed only white colonies on the X-gal plates under either aerobic or anaerobic conditions, indicating a lack of adenylate cyclase activity and, accordingly, no detectable interaction between IspG and IspH from either organism.

## DISCUSSION

Strategies for optimization of the MEP pathway are of significant interest because of the value of isoprenoid-based bioproducts. The terminal steps catalyzed by the [4Fe-4S] cluster containing IspG and IspH enzymes have often been noted as bottlenecks in the pathway (9). Here, we focused on these two enzymes from *Z. mobilis* to begin understanding the attributes that potentially limit their function. Our analysis of *Z. mobilis* IspH and IspG proteins expressed in *E. coli* indicated that neither protein could fully complement the respective *E. coli* mutants even under anaerobic conditions, indicating significant functional differences between the *E. coli* and *Z. mobilis* proteins. Co-expression of *Z. mobilis* IspG and IspH improved the function of IspH under anaerobic conditions, suggesting a functional linkage between the activity of the two enzymes despite our inability to detect any physical association. Finally, *Z. mobilis* IspH caused an oxygen-sensitive growth phenotype that could be partially mitigated by co-expression of cognate IspG, and both enzymes were shown to have O_2_-sensitive [4Fe-4S] cluster cofactors. Overall, these data suggest that both enzymes need to be optimized in parallel to improve MEP pathway flux.

Why *Z. mobilis* IspG poorly complements *E. coli*Δ*ispG* is not obvious from the protein sequence. Overall, *Z. mobilis* IspG is 48.4% identical over the entire length of *E. coli* IspG (Fig. S9). The alpha-fold prediction of a *Z. mobilis* IspG monomer shows an overall structural similarity to the *E. coli* prediction and to either subunit of the dimeric IspG X-ray crystal structure from *T. thermophilus* B8 (35). According to the crystal structure, the protein folds into two domains, a triose-phosphate isomerase (TIM) barrel and an Fe-S cluster domain. The [4Fe-4S] cluster of IspG is ligated by 3 Cys residues and one Glu, and these residues are conserved for both *Z. mobilis* (Cys272, Cys275, Cys307, and Glu 314) and *E. coli* (Cys270, Cys273, Cys305, and Glu 312) IspG. The residues in *T. thermophilus* that bind the substrate, MEcDP, and hold it in place via a hydrogen bond network between the [4Fe-4S] cluster and the TIM barrel (Arg56, Arg110, Arg141, Lys204, and Arg260) are also conserved. The substrate binds directly to the [4Fe-4S] cluster, displacing ligation by Glu350 and brings the conserved Glu232 into proximity of the MEcDP C3 hydroxyl group forming a closed state for catalysis, in which the active site is no longer solvent-exposed (13, 14, 21, 35). It is worth noting that *Z. mobilis* IspG has four additional cysteines in the TIM barrel domain that are not present in *E. coli*, raising the possibility that it has another metal center. Nevertheless, the conservation of the overall structure and the key residues for binding substrate and the Fe-S cluster in all three proteins imply a conserved active site and reaction mechanism for the IspG-catalyzed reductive ring opening to form HMBDP.

It is also not clear from the protein sequence of *Z. mobilis* IspH why this protein complements *E. coli*Δ*ispH* poorly. *Z. mobilis* IspH is 52.5% identical over the entire length of *E. coli* IspH (Fig. S10). The alpha-fold prediction of a *Z. mobilis* IspH monomer shows an overall structural similarity to the X-ray crystal structure of *E. coli* IspH (16). The protein folds as a “trefoil” arrangement consisting of three α/β domains with the Fe-S cluster bound at the center of the structure. IspH belongs to a subclass of Fe-S cluster proteins, illustrated by aconitase, where Cys residues provide three ligands to the [4Fe-4S] cluster, but the fourth Fe ligand of the [4Fe-4S] cluster is provided by the substrate and substrate availability can impact Fe-S cluster occupancy (16, 17, 20, 35–38). The X-ray structure of *E. coli* IspH shows that the C4-OH group of HMBDP displaces bound water to form the fourth Fe ligand to the [4Fe-4S] cluster (37). Cys12, Cys 96, and Cys197 provide the other cluster ligands, and the position of these is conserved in *Z. mobilis* IspH (Fig. S10). E126, which plays a key role during catalysis to bind the CH_2_OH group of HMBDP to promote the conversion to IDP or DMADP (17, 38), is also conserved. Thus, the conservation between *E. coli* and *Z. mobilis* of the overall IspH structure and key residues for binding substrate and the Fe-S cluster imply a conserved active site and reaction mechanism for the IspH-catalyzed reductive dehydroxylation of HMBDP to IDP and DMADP.

**Fig 9:**
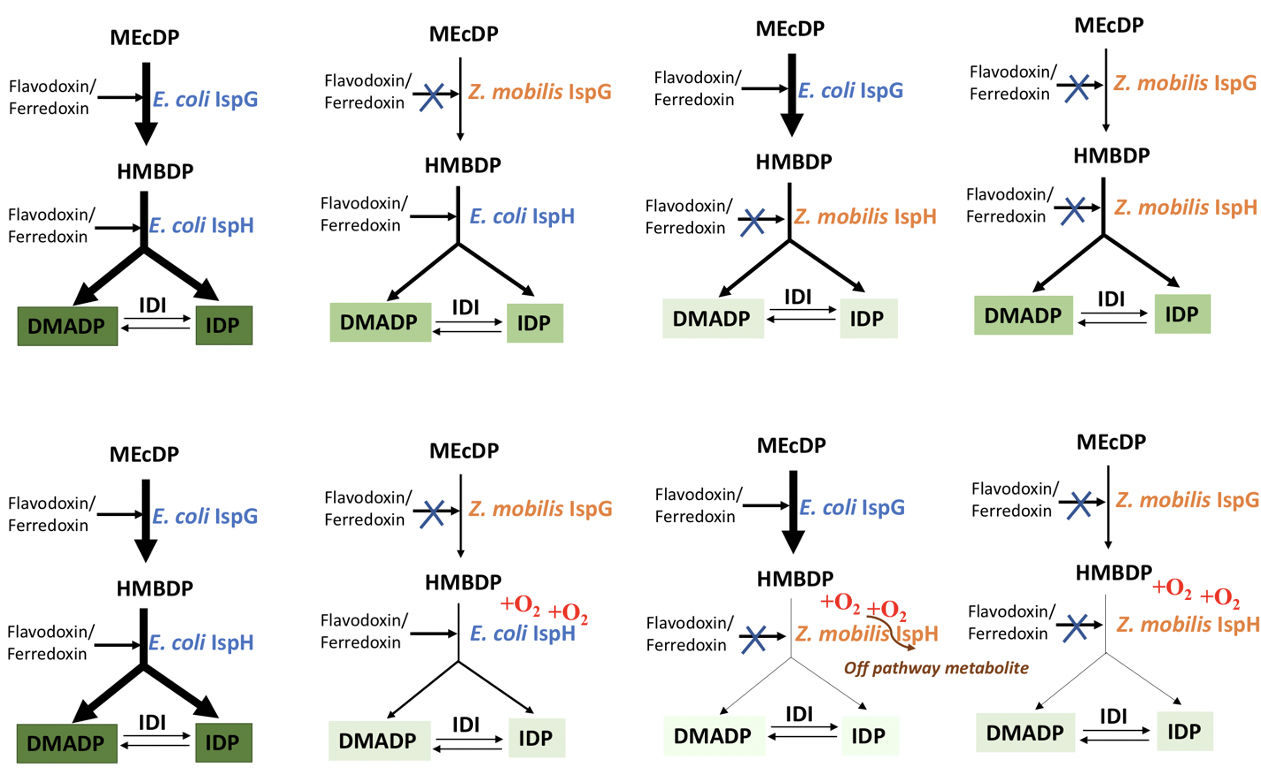
Proposed model describing *Z. mobilis* and *E. coli* IspH and IspG function under anaerobic (top panel) and aerobic (bottom panel) conditions. The arrows represent the proposed flux through the terminal steps of the MEP pathway; thick lines represent high flux, whereas thin lines represent low flux and assumes nonoptimal interactions with redox proteins (flavodoxin or ferredoxin) indicated by X. The shade of green indicates the relative amount of DMADP and IDP produced, with dark green being the most and light green being the least.

Although we cannot rule out differences in *K*_M_ or *k*_cat_ contributing to the poor function of *Z. mobilis* IspG and IspH in *E. coli* since these values are unknown, another gap in our knowledge is the identity of the physiological redox partners that serve as the electron donors for these reactions (Fig. 9). Electron transfer from redox proteins to the [4Fe-4S] cluster of both IspG and IspH is required for each catalytic cycle (35, 36). In plants, reduced ferredoxin (FdxA) supplies electrons to the IspG [4Fe-4S] cluster, whereas in *E. coli*, electrons are supplied through flavodoxin I (FldA) (39, 40). The electron transfer proteins involved in reducing the [4Fe-4S] cluster of IspG and IspH in *Z. mobilis* are unknown. From the genome annotation of *Z. mobilis* in KEGG*,* there are five predicted ferredoxins (ZMO1818, ZMO0220, ZMO2028, ZMO0860, and ZMO0456) and one predicted flavodoxin (ZMO1851) and a second possible flavodoxin (ZMO1949) that are possible candidates for these electron transfer reactions. A recent study suggests that organisms with multiple protein electron donors are more likely to have evolved “specialists” by optimizing binding specificity and electron transfer rates for specific enzyme partners and extended protein regions that exclude other proteins (41).

In support of species-specific redox partners for IspG, a recent study showed that the majority of orthologous IspG proteins surveyed from phylogenetically representative bacteria failed to complement *E. coli*Δ*ispG* strains (22). For a small subset, the lack of complementation could be explained by a preference for their native electron transfer proteins through co-expression with its native IspG in *E. coli*. The least conserved region between *Z. mobilis* and *E. coli* IspG is at the C-terminus. Since there is no structural or biochemical information to indicate where flavodoxin or ferredoxin dock on any IspG, such variation at the C-termini could be significant in specifying a particular redox partner and thus affect function. Thus, future efforts for optimization of the MEP pathway in *Z. mobilis* should focus on identifying which accessory electron transfer proteins are required for *Z. mobilis* IspG and IspH function and determine if their co-expression with IspG and IspH improves MEP pathway function in *E. coli* or *Z. mobilis*.

A similar scale survey of heterologously expressed IspH orthologs in *E. coli* has not been undertaken to our knowledge. However, a phylogenetic analysis indicates considerable diversity of IspH at their N and C termini (20). Biochemical and bioinformatic analysis led to the proposal that there are four main classes of IspH, and this variation may influence the stability of the Fe-S cluster in aerobic conditions. *E. coli and Z. mobilis* IspH belong to class B because of the aromatic residues found near the [4Fe-4S] cluster of IspH as well as longer C-terminal extensions. In fact, the C-terminal extension of *Z. mobilis* IspH is longer than that of *E. coli* IspH, which could affect enzyme activity or interaction with a redox partner. We reason that perhaps because *Z. mobilis* is an anaerobic bacterium that is aerotolerant, IspH has not experienced sufficient selective pressure to evolve to protect its active site and [4Fe-4S] cluster against transitions to aerobic growth.

We also found functional connections between IspG and IspH from *Z. mobilis* since coexpression of *Z. mobilis ispH* with *ispG* improved the complementation of *E. coli*Δ*ispH*. However, our results do not yet support a physical association since all three assays (molecular sieve analysis, native gel electrophoresis, and BACTH two-hybrid screen) failed to detect an interaction between the two proteins under the conditions tested. However, we also acknowledge that an interaction might be too transient to detect, that the physiological electron donors may be required, and/or that an interaction is influenced by the presence of the pathway substrates. Since IspG catalyzes the conversion of MEcDP to HMBDP and HMBDP is a ligand to the [4Fe-4S] cluster of IspH, a reasonable expectation is that cluster stability and occupancy of IspH is linked to the availability of its substrate HMBDP, connecting it to the function of IspG. In summary, detection of protein:protein interactions might require the appropriate electron transfer partners, as well as substrates and highly occupied clusters containing IspG and IspH.

Under aerobic conditions, *Z. mobilis* IspH prevented the growth of *E. coli*Δ*ispH* even in the presence of mevalonate, suggesting that the enzyme was making an inhibitory product (Fig. 9). We considered this might be due to accumulation of HMBDP in these strains, since a previous study suggested that accumulation of HMBDP could be inhibitory to cells (42). However, we found that IspH activity was required for this inhibition, since a catalytically inactive *Z. mobilis* variant no longer prevented growth in the presence of mevalonate. We hypothesize that the open conformation of the *Z. mobilis* IspH active site, which is known for *E. coli* IspH to be more prone to [4Fe-4S] cluster oxidation and degradation (20, 35), may be favored under aerobic conditions. Whether this results from a lack of interaction with its cognate IspG and a resulting decrease in HMBDP binding to stabilize the [4Fe-4S] cluster is unknown. It is worth noting that the presence of *Z. mobilis* IspG and not *E. coli* IspG suppresses this inhibitory activity, favoring the notion of a specific interaction of *Z. mobilis* IspH with *Z. mobilis* IspG. If the lack of a matching ferredoxin or flavodoxin is also limiting, then the oxidized cluster would be less likely to be reduced to the functional [4Fe–4S]^1+^ form. IspH is known to have acetylene hydratase activity and catalyze the conversion of acetylenes to aldehyde and ketone (19). We speculate that such a promiscuous activity produces a compound that inhibits growth. Future work will be needed to address this hypothesis.

A previous study also reported that balanced activities of IspG and IspH are important for MEP pathway function. If IspH levels were not sufficient, then HMBDP accumulated, which the authors suggest could be inhibitory to cells (42). In this study, we observed optimal MEP pathway function when *ispG* and *ispH* were co-expressed in a strain lacking only IspH, compared with expression of *ispH* alone. However, this increase in viability cannot easily be explained by HMBDP levels, since more HMBDP would be expected in the co-expression strain because it also carries a functional *E. coli ispG* on the chromosome. Furthermore, under aerobic conditions, the remarkable preference for the cognate IspH and IspG pairs in strains co-expressing different combinations of *Z. mobilis* and *E. coli* IspG and IspH deleted for *ispG* and *ispH* on the chromosome can only be reasonably explained by specific protein:protein interactions between these proteins and not just the capacity to make HMBDP (Fig. 9).

The Fe-S cluster maturation machinery does not appear to be limiting for *Z. mobilis* IspG and IspH function in *E. coli*. Previous studies showed that *E. coli* isoprenoid synthesis relies on the ISC housekeeping Fe-S maturation pathway (22, 30). Moreover, other studies examined the impact of levels of Fe-S cluster machinery on IspH or IspG activity expressed in *E. coli* (22, 43). *Z. mobilis* has only the SUF Fe-S biogenesis pathway (9) raising the question whether a preference for an orthologous pathway limited function in *E. coli*. However, the fact that increased expression of either the *E. coli* or *Z. mobilis* SUF Fe-S cluster maturation pathway did not improve *Z. mobilis* IspG or IspH functions suggested there was no preference for Fe-S cluster biogenesis pathways, and this was not the source of limited function.

In summary, the results presented here reveal a species specificity of IspG and IspH function that is particularly striking under aerobic growth conditions. The knowledge gained here is central to understanding the function of *Z. mobilis* IspG and IspH under aerobic and anaerobic conditions. Identifying the physiological electron donors and determining the molecular basis of the species specificity will provide insight to engineering the steps catalyzed by these unique [4Fe-4S] containing enzymes and improve the flux through the MEP pathway to produce isoprenoid precursors.

## SUPPLEMENTAL MATERIAL

Supplemental material spectrum.04256-23-s0001.pdf Fig. S1 to S10; Table S1.

ASM does not own the copyrights to Supplemental Material that may be linked to, or accessed through, an article. The authors have granted ASM a non-exclusive, world-wide license to publish the Supplemental Material files. Please contact the corresponding author directly for reuse.
