# Supplementary material for: Functional analysis of the methylerythritol phosphate pathway terminal enzymes IspG and IspH from Zymomonas mobilis
Source: Microbiol Spectr. 2024 May 24;12(7):e04256-23. doi: 10.1128/spectrum.04256-23 (PMC11218510; doi:10.1128/spectrum.04256-23)
Supplement: Supplemental material — Fig. S1 to S10; Table S1. [file spectrum.04256-23-s0001.pdf]

Supplemental Data

Functional analysis of the methylerythritol phosphate pathway terminal enzymes IspG and IspH from *Zymomonas mobilis*

Jyotsna Misra[1,2], Erin L. Mettert[1], Patricia J. Kiley[1,2*]

[1]Department of Biomolecular Chemistry, University of Wisconsin-Madison, Madison, WI, USA

[2]DOE Great Lakes Bioenergy Research Center, University of Wisconsin-Madison, Madison, WI, United States

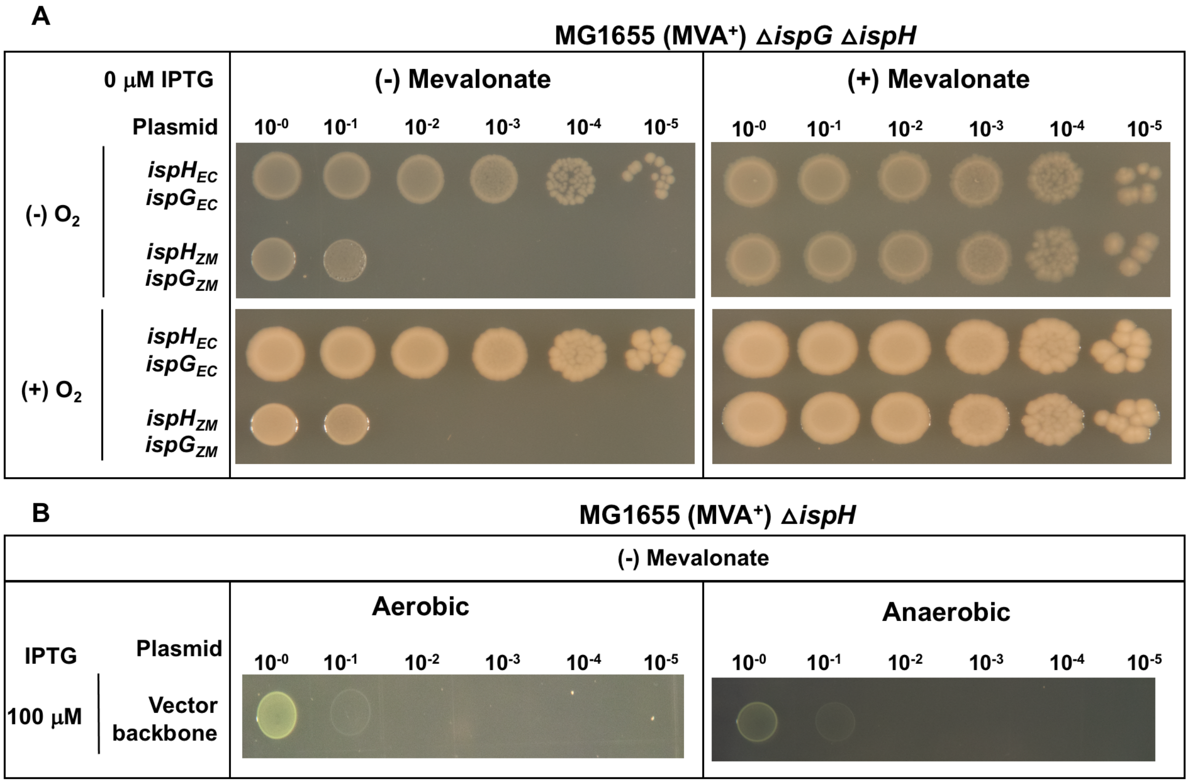

Figure S1. (A) MG1655 MVA$^+$ ∆*ispG* ∆*ispH* with plasmid variants containing *ispG* and *ispH* from *Z. mobilis* (*ispH$_{ZM}$ ispG$_{ZM}$*) or *E. coli* (*ispH$_{EC}$ ispG$_{EC}$*) were grown in LB with mevalonate, arabinose and spectinomycin and plated with no IPTG (B) MG1655 MVA$^+$ ∆*ispH* with plasmid backbone pRL814 plated in the absence of mevalonate. Viability of cells was assayed as described in Fig.1

A

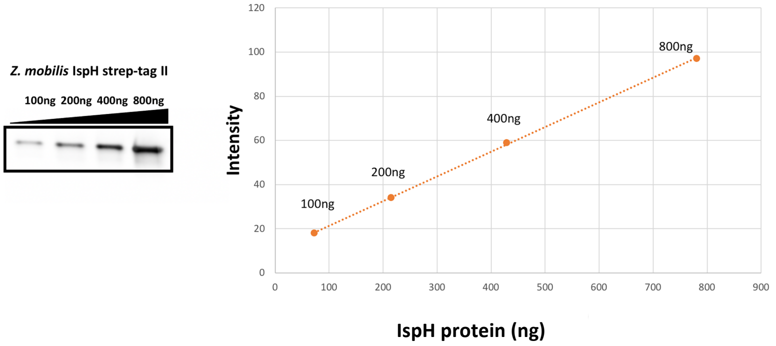

B

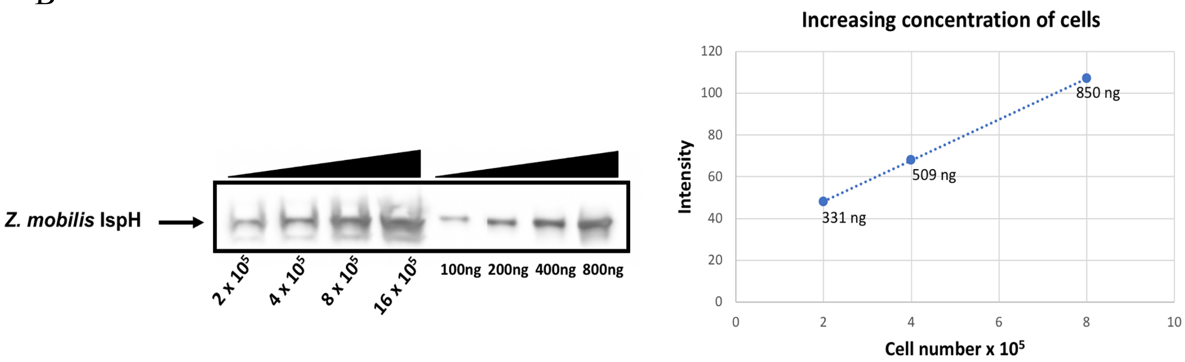

Figure S2. Detection of IspH in cell extracts by Western blots. (A) Establishing a linear range of detection of *Z. mobilis* IspH-Strep tag II with the strep-tag II antibody. Increasing concentrations of isolated *Z. mobilis* IspH with strep-tag II at C terminus, separated on SDS-PAGE, transferred to nitrocellulose membrane and probed with antibody against strep-tag II. The blot was imaged using an Azure imager, quantified using the AzureSpot Pro software and established a linear range of detection between 100-800 ng protein concentration. **(B)** Establishing a cell number where detection of plasmid encoded *Z. mobilis* IspH strep-tag II in MG1655 (MVA^+) Δ*ispH* is within the linear range of detection after Western blotting with antibody against strep-tag II. The strain was grown aerobically with 100 μM IPTG. Cell number was varied as indicated and the blot was processed and quantified as in **A**.

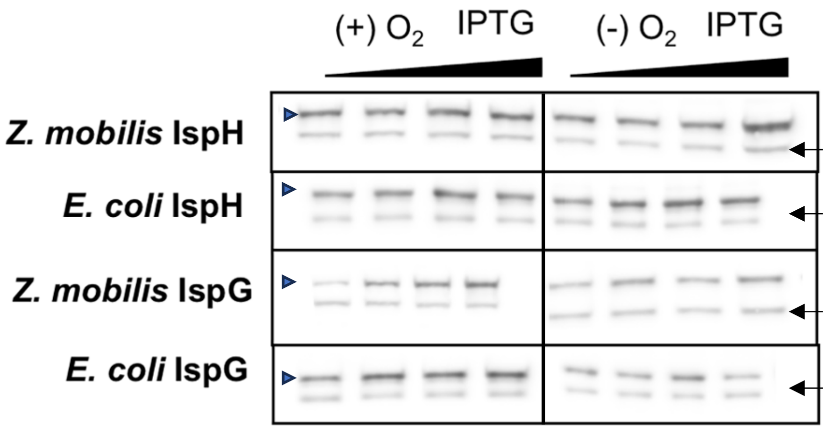

Figure S3. Cultures of MG1655 (MVA^+) ∆*ispH* with *Z. mobilis* IspH strep-tag II at C-terminus **(top panel),** MG1655 (MVA^+) ∆*ispH* with *E. coli* IspH strep-tag II at C-terminus **(second panel),** MG1655 (MVA^+) ∆*ispG* with *Z. mobilis* IspG strep-tag II at C-terminus **(third panel)** MG1655 (MVA^+) ∆*ispG* with *E. coli* IspG strep-tag II at C-terminus (**fourth panel**) were grown with increasing IPTG concentrations (25μM, 50μM, 100μM, or 200μM) under aerobic and anaerobic conditions. Cell pellets were resuspended in 1X SDS sample buffer, heated to 95°C, separated by SDS-PAGE alongside IspH-strep tag II standards, transferred to nitrocellulose membrane and probed with antibody against strep-tag II. Representative blots are shown. The top band (triangle) in each lane is the strep tagged protein- either IspG or IspH. The bottom band (arrow) represents a cross reacting protein with antibody against strep-tag II.

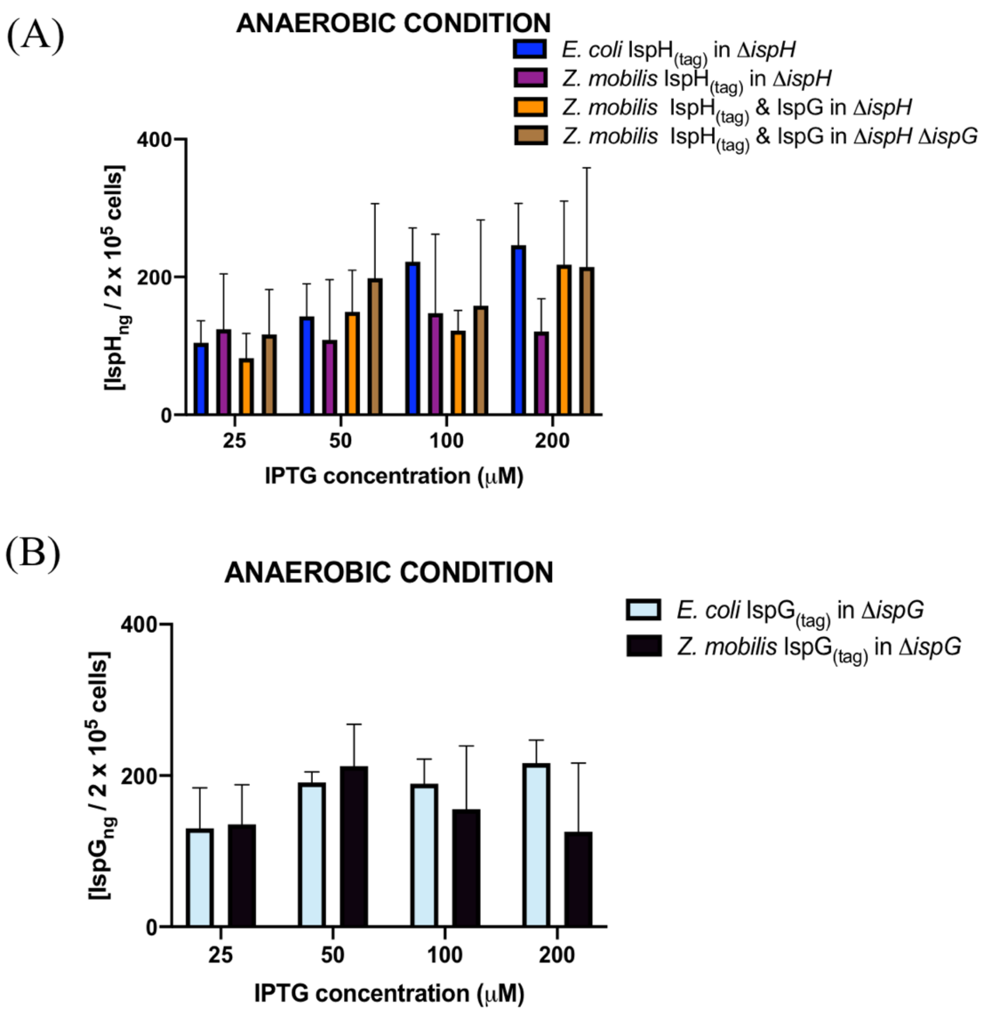

Figure S4. Quantification of Western blots of anaerobically grown cultures described in Figure S3. Blots were imaged using an Azure imager and quantified as described in Figure S2. **(A)** Amount of IspH-Strep-tag II protein normalized to cell number. **(B)** Amount of IspG-Strep-tag II protein normalized to cell number. Data represent averages of protein levels from at least three replicates. Error bars show standard error of the mean.

(A)

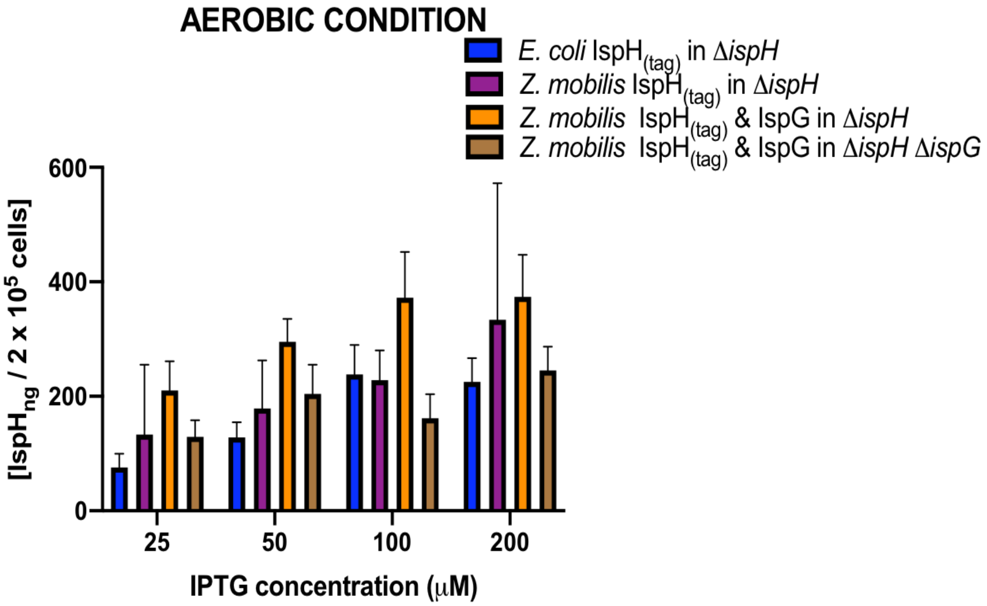

(B)

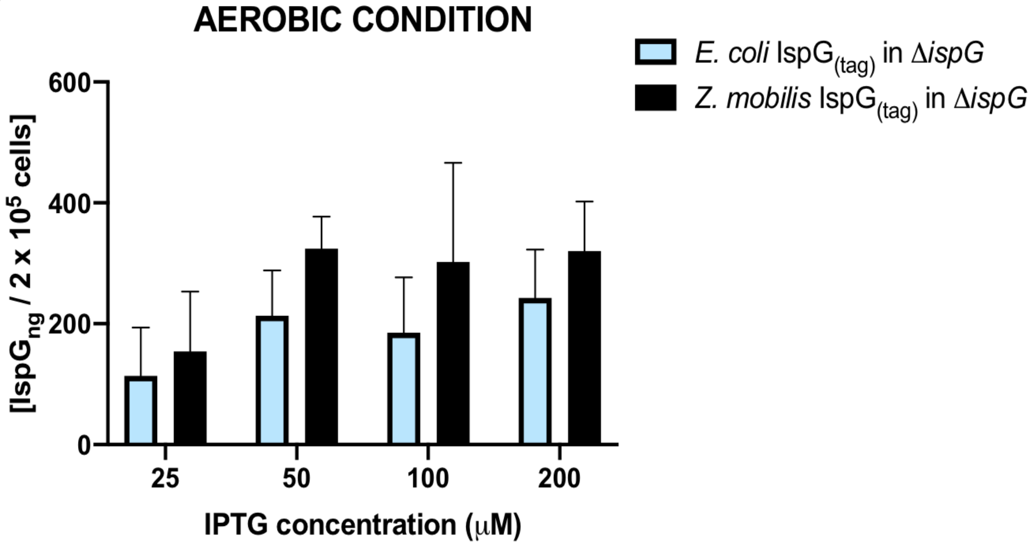

Figure S5. Quantification of Western blots of aerobically grown cultures described in Figure S3. Blots were imaged using an Azure imager and quantified as described in Figure S2. **(A)** Amount of IspH-Strep-tag II protein normalized to cell number. **(B)** Amount of IspG-Strep-tag II protein normalized to cell number. Data represent averages of protein levels from at least three replicates. Error bars show standard error of the mean.

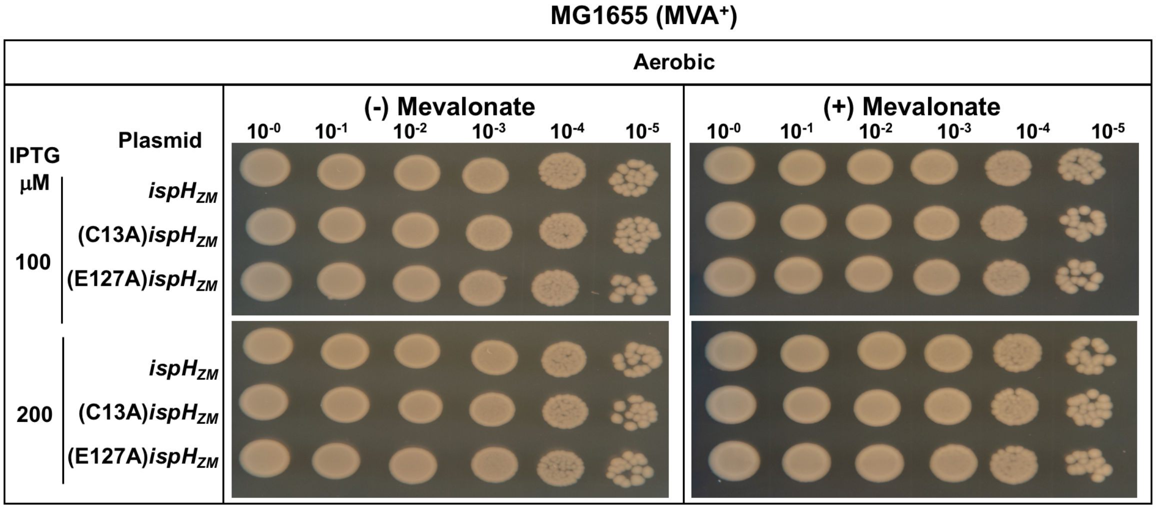

Figure S6. MG1655 (MVA$^+$) with plasmid variants containing *ispH* from *Z. mobilis* (*ispH$_{ZM}$)* or with point mutations E127A or C13A were grown in LB with mevalonate, arabinose and spectinomycin. Viability of cells was assayed as described in Figure 1

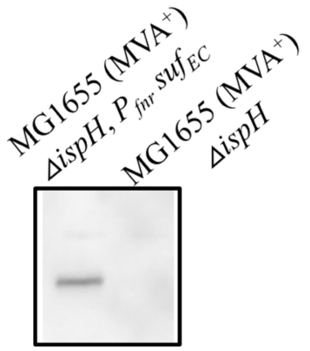

Figure S7. Comparison of SufD levels in MG1655 (MVA$^+$) Δ*ispH* to MG1655 (MVA$^+$) Δ*ispH* P_{fnr} suf_{EC} grown under aerobic conditions. Equivalent amount of cell lysates was separated by SDS-PAGE, transferred to nitrocellulose membrane and probed with antibody against SufD. The blot was imaged using an Azure imager.

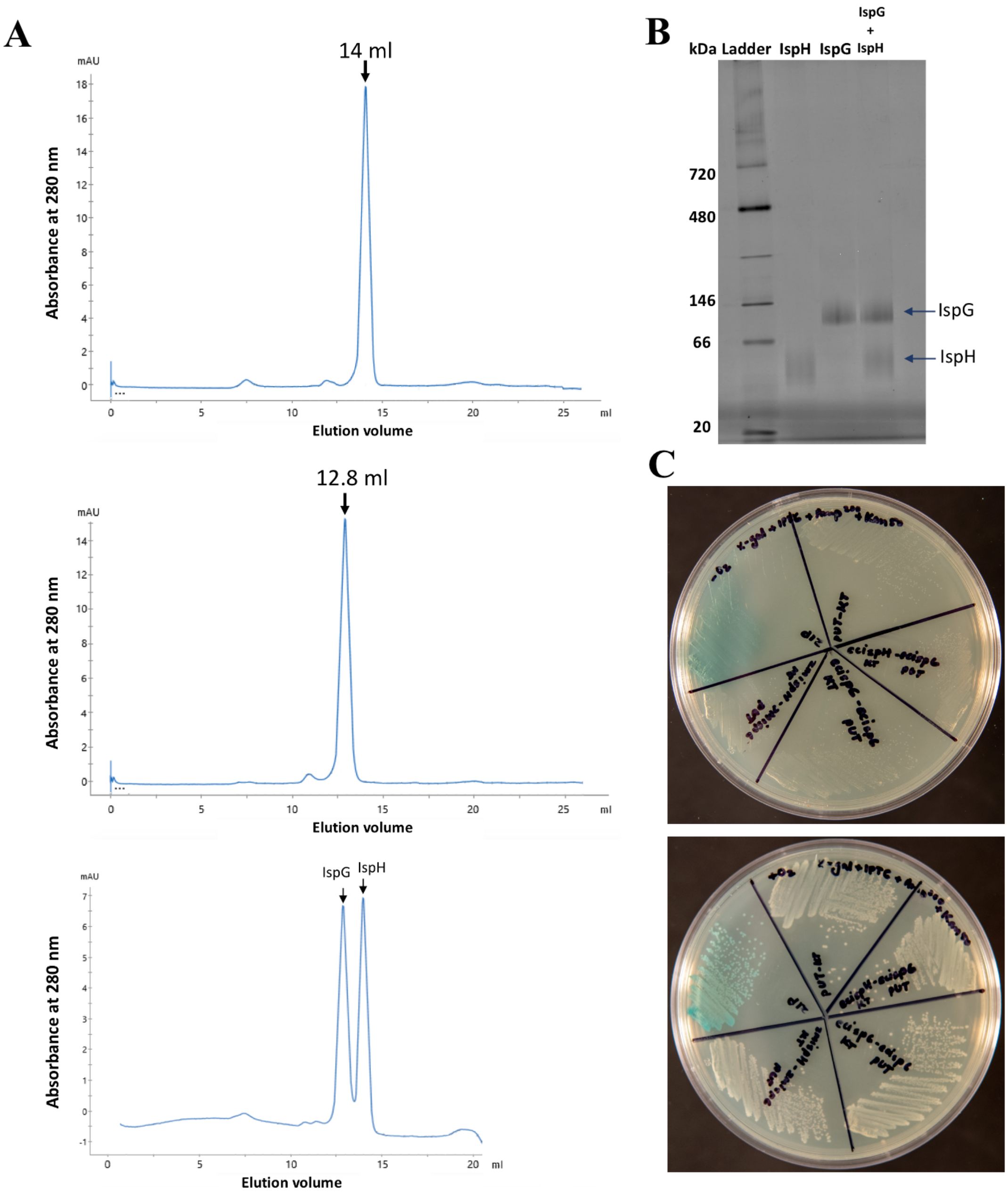

Figure S8. (A) The anaerobic elution profiles of *Z. mobilis* Strep-tag II IspH (top panel), Strep-tag II IspG (middle panel) and an equimolar mixture of *Z. mobilis* Strep-tag II IspG and Strep-tag II IspH (bottom panel) from a Superdex 200 Increase 10/300 GL column. (B) Native Blue Gel electrophoresis of standard molecular weight markers, *Z. mobilis* Strep-tag II IspH, Strep-tag II IspG, and an equimolar mixture of Strep-tag II IspG & Strep-tag II IspH under aerobic conditions. (C) BTH 101 with plasmid variants (T25-*ispH*$_{ZM}$ & T18-*ispG*$_{ZM}$), (T25-*ispH*$_{EC}$ & T18-*ispG*$_{ZM}$), (T25-*ispG*$_{EC}$ & T18-*ispG*$_{EC}$), (T25 & T18), (T25-*zip* & T18-*zip*) grown under aerobic and anaerobic conditions. Anaerobic plates were exposed to oxygen to allow the oxygen dependent dimerization of the dye to form a blue color.

```
E.coli        --MHNQAPIQRRKSTRIYVGNVPIGDGAPIAVQSMTNTRTTDVEATVNQIKALERVGADI     58
Z.mobilis     MSIRPWRHIERRKSRKIMVGNVAVGGDAPISVQTMTNTPTVDAQATIAQIKRCEAVGVDL     60
                ::    *:**** :* **** :*..***:**:**** *.*.:**: ***  * **.*:

E.coli        VRVSVPTMDAAEAFKLIKQQVNVPLVADIHFDYRIALKVAEYGVDCLRINPGNIGNEERI     118
Z.mobilis     IRVSCPDKESTAALKDIVRAAEVPIIADIHFHYKRALEAADAGAACLRINPGNIGSSERV     120
              :*** *  ::: *:* * : .:**::*****.*: **:.*: *. **********..**:

E.coli        RMVVDCARDKNIPIRIGVNAGSLEKDLQEKYGEPTPQALLESAMRHVDHLDRLNFDQFKV     178
Z.mobilis     AEVVRAAKANGCAIRIGVNAGSLEKELLEKYGEPCPDALVESALNHIKLLQDQDFHEFKV     180
                ** .*: :.  ************:* ****** *:**:***:.*:. *:  :*.:***

E.coli        SVKASDVFLAVESYRLLAKQIDQPLHLGITEAGGARSGAVKSAIGLGLLLSEGIGDTLRV     238
Z.mobilis     AVKASDVFLAVASYKALAKAVDCPLHLGITEAGGLIGGTVKSALGIGNLLWDGIGDTLRV     240
              :********** **: *** :* ***********  .*:****:*:* ** :********

E.coli        SLAADPVEEIKVGFDILKSLRIRSRGINFIACPTCSRQEFDVIGTVNALEQRLEDIITPM     298
Z.mobilis     SLSADPEQEVRVGYDILKTLDLRTRGVRVVSCPSCARQGFDVVKTVKALEERLAHIATPI     300
              **:*** :*::**:****:* :*:**:..::**:*:** ***: **:***:** .* **:

E.coli        DVSIIGCVVNGPGEALVSTLGVTGGNKK-SGLYEDGVRKDRLDNNDMIDQLEARIRAKAS     357
Z.mobilis     SLSILGCVVNGPGEARETDIGVTGGGQGKHMVFLSGVTDHTVEDAKMLDHIVSLVEAKAA     360
              .:**:*********  : :*****.:    :: .** .. ::: .*:*:: : :.***:

E.coli        QLDEARRIDVQQVEK--          372
Z.mobilis     EIEAEKAKEKAATVAAE          377
              :::  :  :   .
```

Figure S9. Sequence alignment of *E. coli* and *Z. mobilis* IspG. The residues (Cys, Glu) that ligate the [4Fe-4S] cluster are boxed in red and the catalytic Glu is in purple. The percent identity between the two sequences is 48.4%.

```
E.coli          -MQILLANPRGFCAGVDRAISIVENALAIYGAPIYVRHEVVHNRYVVDSLRERGAIFIEQ     59
Z.mobilis       MIKIILAQPRGFCAGVIRAIEIVDQALDRVGAPVYYVRHEIVHNRHVVDTLKAKGAVFVEE    60
                 ::*:**:******** ***.**::**   ***:*****:****:***:*: :**:*:*:

E.coli          ISEVPDGAILIFSAHGVSQAVRNEAKSRDLTVFDATCPLVTKVHMEVARASRRGEESILI     119
Z.mobilis       LSEVPPGEVTVFSAHGVARTVQKEAEERGLPVVDATCPLVNKVHAQGRRYLSHGRTLILI     120
                :**** * : :******:::*::**:.*.* *.*******.*** :  *   :*.  ***

E.coli          GHAGHPEVEGTMGQYSNPEGGMYLVESPDDVWKLTVKNEEKLSFMTQTTLSVDDTSDVID     179
Z.mobilis       GHAGHPEIEGTLGQIDGP---VHLVGSAEQVDSLDIPSDTPVAFITQTTLSVDDTRSVIA     177
                *******:***:** ..*   ::** * ::* .* : .:  ::*:********** .**

E.coli          ALRKRFPKIVGPRKDDICYATTNRQEAVRALAEQAEVVLVVGSKNSSNSNRLAELAQRMG     239
Z.mobilis       ALRKKFTDVIGPDTSDICYATQNRQAAVRDLCKQSDLILVVGSPNSSNSNRLREIGLEEG     237
                ****:* .::** ..****** *** *** *.:*::::***** ******** *:. . *

E.coli          KRAFLIDDAKDIQEEWVKEVKCVGVTAGASAPDILVQNVVARLQQLGGGEAIPLEGREEN     299
Z.mobilis       LPSYLIADGSEIDPVWFEGINTVGLTAGASAPEELVQSVITAIRALGPVTVEQLDGVEEK     297
                  ::** *..:*:  *.: :: **:*******: ***.*:: :: **   .  *:* **:

E.coli          IVFEVPKELRVDIREVD--------------------------      316
Z.mobilis       IAFRLPPTLRHLKARNAAHNNLDNKTAASEEADSLSNDTEQEA      340
                *.*.:*  **    .
```

Figure S10. Sequence alignment of *E. coli* and *Z. mobilis* IspH. The residues (Cys) that ligate the [4Fe-4S] cluster are boxed in red and the catalytic Glu is in purple. The percent identity between the two sequences is 52.5%.

Table S1: Primers used in this study

Primer number	Primer sequence (5'-3')
1a	CTCCTTCTTAAG**AAAGATTCAGGTTTCATACAAAGGAGGACGGAT**ATGCAGATCCTGTTGGCCAACC
1b	GCGTGTCGACTTAATCGACTTCACGAATATC
1c	CTCCTTCTTAAGAAAGATTCAGGT
2a	CTCCTTCTTAAG**AAAGATTCAGGTTTCATACAAAGGAGGACGGAT**GTGATAAAAATCATTCTGGCTC
2b	GCGTGTCGACTCAAGCCTCCTGTTCTGTATC
2c	CTCCTTCTTAAGAAAGATTC
3a	CTCCTTCTTAAG**CTGAAATTAGTTTAGGAGAAAGAAT**ATGCATAACCAGGCTCCAATTC
3b	GCGTGTCGACTTATTTTTCAACCTGCTGA
3c	CTCCTTCTTAAGCTGAAATT
4a	CTCCTTCTTAAG**CTGAAATTAGTTTAGGAGAAAGAAT**ATGTCCATTCGTCCTTGGCGCC
4b	GCGTGTCGACTTATTCGGCGGCGACCGTGGC
4c	CTCCTTCTTAAGCTGAAATT
5a	TGCTGGAAATCGATCCGGCACTGGAGGCGTAACATGATTCCGGGGATCCGTCGACC
5b	GTATTTTCGCATAACTTAGGCTGCTAATGACTTAATGTGTAGGCTGGAGCTGCTTC
6a	GCAGTAACAGACGGGTAACGCGGGAGATTTTTCATGATTCCGGGGATCCGTAGACC
6b	CACGGGAAGCGAGGCGCTTCCCATCACGTTATTATTGTGTAGGCTGGAGCTGCTTC
7a	GCTTGATATCGAATTCCTG
7b	TTACTCGAGTTTGTAGAGC
8a	AGCTCTACAAACTCGAGTAACTGAAATTAGTTTAGGAGAAAGAATATGTCCATTCG
8b	GCAGGAATTCGATATCAAGCTTATTCGGCGGCGACCGT
9a	AGCTCTACAAACTCGAGTAACTGAAATTAGTTTAGGAGAAAG
9b	GCAGGAATTCGATATCAAGCTTATTTTTCAACCTGCTGAAC
10a	GCCGAATAAGCTTGATATCGAATTCCTGCAGC
10b	AACTAATTTCAGAGTTAAATCGACTTCACGAATATCGACAC
11a	AGTCGATTAACTCTGAAATTAGTTTAGGAGAAAGAATATGTCCATTCGT
11b	GATATCAAGCTTATTCGGCGGCGACC
12a	GTTGAAAAATAAGCTTGATATCGAATTCCTGCAGC
12b	CTAATTTCAGACTCAAGCCTCCTGTTCTGTATCG
13a	GCTTGAGTCTGAAATTAGTTTAGGAGAAAGAATATGCATAACCAGG
13b	TCGATATCAAGCTTATTTTTCAACCTGCTG
14a	CATCCTCAATTTGAAAAATAACGACACAATCTGTCCTTTC
14b	CGACCAGCCGCCAAAGCAGCCAGCCTCCTGTTCTGTATC
15a	CATCCTCAATTTGAAAAATAACGACACAATCTGTCCTTTC
15b	CGACCAGCCGCCAAAGCAGCCATCGACTTCACGAATATCGAC
16a	CATCCTCAATTTGAAAAATAACGACACAATCTGTCCTTTCG
16b	CGACCAGCCGCCAAAGCAGCCTTCGGCGGCGACC

17a	CATCCTCAATTTGAAAAATAACGACACAATCTGTCCTTTCG
17b	CGACCAGCCGCCAAAGCAGCCTTCGGCGGCGACC
18a	AGATATACATGTGATAAAAATCATTCTGGC
18b	CTTTCGGGCTTTATTTTTCAAATTGAGGATGC
19a	TGAAAAATAAAGCCCGAAAGGAAGCTGAG
19b	TTTTTTATCACATGTATATCTCCTTCTTAAAGTTAAACAAAATTATTTCTAG
20a	AGATATACATATGTCCATTCGTCCTTGG
20b	CTTTCGGGCTTTATTTTTCAAATTGAGGATGCG
21a	AGATATACATATGCAGATCCTGTTGGCCAAC
21b	CTTTCGGGCTTTATTTTTCAAATTGAGGATGCGACCAG
Z. mobilis IspG pUT F	AGGATCCCGGGTACTCCATTCGTCCTTGGCGCCATATAGA
Z. mobilis IspG pUT R	ATCCTCTAGAGTCGACCTGCAGTGGCGTTCCACTGCG
Z. mobilis IspH KT F	ATCCCGGGTACATAAAAATCATTCTGGCTCAACCGC
Z. mobilis IspH KT R	ATCCTCTAGAGTCGACCTGCAGTGGCGTTCCACTGCG
E. coli IspG KT F	ATCCCGGGTACCATAACCAGGCTCCAATTCAACG
E. coli IspG KT R	ATCCTCTAGAGTCGACCTGCAGTGGCGTTCCACTGCG
E. coli IspG pUT F	ATCCCCGGGTACCATAACCAGGCTCCAATTCAAC
E. coli IspG KT R	ATCCTCTAGAGTCGACCTGCAGTGGCGTTCCACTGCG
E. coli IspH KT F	ATCCCCGGGTACCAGATCCTGTTGGCCAACC
E. coli IspH KT R	ATCCTCTAGAGTCGACCTGCAGTGGCGTTCCACTGCG
